# Pin1 promotes human Ca_V_2.1 channel polyubiquitination by RNF138: pathophysiological implication for episodic ataxia type 2

**DOI:** 10.1186/s12964-024-01960-9

**Published:** 2024-11-28

**Authors:** Ssu-Ju Fu, Kai-Min Cheng, Cheng-Tsung Hsiao, Ya-Ching Fang, Chung-Jiuan Jeng, Chih-Yung Tang

**Affiliations:** 1 Department of Physiology, College of Medicine, National Taiwan University, Taipei, 100 Taiwan; 2 Department of Neurology, Taipei Veterans General Hospital, Taipei, 112 Taiwan; 3 Department of Neurology, School of Medicine, National Yang Ming Chiao Tung University, Taipei, 112 Taiwan; 4 Institute of Anatomy and Cell Biology, College of Medicine, National Yang Ming Chiao Tung University, Taipei, 112 Taiwan; 5 Brain Research Center, National Yang Ming Chiao Tung University, Taipei, 112 Taiwan

**Keywords:** Peptidyl-prolyl *cis/trans* isomerase, E3 ubiquitin ligase, Proteasomal degradation, Proteostasis, ER quality control, Channelopathy

## Abstract

Loss-of-function mutations in the human gene encoding the neuron-specific Ca^2+^ channel Ca_V_2.1 are linked to the neurological disease episodic ataxia type 2 (EA2), as well as neurodevelopmental disorders such as developmental delay and developmental epileptic encephalopathy. Disease-associated Ca_V_2.1 mutants may exhibit defective proteostasis and promote endoplasmic reticulum (ER)-associated degradation of their wild-type (WT) counterpart in a dominant-negative manner. The E3 ubiquitin ligase RNF138 was previously shown to mediate EA2-related aberrant degradation of Ca_V_2.1 at the ER. Herein we aimed to elucidate the ER proteostasis mechanism of Ca_V_2.1. The peptidyl-prolyl *cis/trans* isomerase, NIMA-interacting 1 (Pin1) was identified as a novel neuronal Ca_V_2.1 binding partner that promoted polyubiquitination and proteasomal degradation of Ca_V_2.1. Suppression of endogenous Pin1 level with either shRNA knockdown or the Pin1 inhibitor all-*trans* retinoic acid (ATRA) enhanced endogenous Ca_V_2.1 protein level in neurons, and attenuated ER-associated degradation of Ca_V_2.1 WT and EA2-causing mutants. Detailed mutation analyses suggested that Pin1 interacted with specific phosphorylated serine/threonine-proline motifs in the intracellular II-III loop and the distal carboxy-terminal region of human Ca_V_2.1. We further generated Pin1-insensitive Ca_V_2.1 constructs and demonstrated that, during ER quality control, Pin1 served as an upstream regulator of Ca_V_2.1 polyubiquitination and degradation by RNF138. Pin1 regulation was required for the dominant-negative effect of EA2 missense mutants, but not nonsense mutants, on Ca_V_2.1 WT protein expression. Our data are consistent with the idea that Ca_V_2.1 proteostasis at the ER, as well as dominant-negative suppression of disease-causing loss-of-function mutants on Ca_V_2.1 WT, entail both Pin1/RNF138-dependent and -independent mechanisms.

**Supplementary Information:**

The online version contains supplementary material available at 10.1186/s12964-024-01960-9.

## Introduction

Voltage-gated P/Q-type Ca^2+^ (Ca_V_2.1) channels are composed of the voltage-sensing pore-forming α_1A_ (Ca_V_2.1) subunit and the auxiliary α_2_δ and β subunits [12, 14]. Ca_V_2.1 channels are widely present in axonal terminals and dendrosomatic regions in the nervous system, and critically contribute to essential neurophysiological processes such as axonal neurotransmitter release, dendritic Ca^2+^ transients, and dendrosomatic membrane excitability [13, 20, 31, 67]. Loss-of-function mutations in the human Ca_V_2.1 subunit-encoding *CACNA1A* gene are linked to the autosomal dominant cerebellar disease episodic ataxia type 2 (EA2), manifesting as paroxysmal attacks of ataxia and nystagmus [36, 37, 63, 67, 71]. More than 100 EA2-causing familial or sporadic *CACNA1A* mutations have been identified, including genomic rearrangement, aberrant splicing, and nonsense and missense mutations [6, 61, 76, 78, 84]. Many of these disease-causing mutations instigate substantial deletion, frame shift, or truncation of the *CACNA1A* gene, resulting in non-functional protein. In addition, EA2 missense mutations are associated with altered Ca_V_2.1 channel gating/permeation function, as well as defective protein homeostasis (proteostasis) characterized by enhanced protein degradation and/or impaired membrane trafficking [24, 30, 34, 39, 59, 70, 83, 85].

In light of the loss-of-function nature of EA2 mutants, haploinffuciency is likely to contribute to the pathogenic mechanism underlying the dominant inheritance of the cerebellar disease [27, 58, 78, 80, 84]. However, some EA2-causing nonsense and missense mutants may exert dominant-negative effect on the proteostasis of their wild-type (WT) counterpart [18, 21, 24, 38, 39, 42, 59, 64, 65, 69]. Emerging evidence suggests that a similar dominant-negative effect on human Ca_V_2.1 proteostasis may also apply to loss-of-function *CACNA1A* mutations associated with developmental epileptic encephalopathy [41].

The detailed mechanisms underlying the dominant-negative effects of disease-causing mutants on WT Ca_V_2.1 proteostasis remain elusive. At least for misfolded EA2 nonsense mutants, the amino terminal region appears to take part in interacting with and eliciting dominant-negative suppression of Ca_V_2.1 WT channel [18, 65], probably causing translational arrest of WT Ca_V_2.1 and other members of the Ca_V_2 channel family via unfolded protein response [64]. Moreover, EA2 nonsense and missense mutants may disrupt protein folding of their WT counterpart, leading to enhanced endoplasmic reticulum (ER)-associated degradation and defective membrane trafficking [24, 39, 59, 69]. We previously addressed the mechanism of Ca_V_2.1 ubiquitin-proteasome degradation pathway by demonstrating that the really interesting new gene (RING) finger E3 ubiquitin ligase RNF138 regulates polyubiquitination and protein stability of Ca_V_2.1, and contributes to aberrant protein degradation of Ca_V_2.1 WT caused by EA2 mutants [24]. Nonetheless, it is still unclear how EA2 mutants can induce enhanced ER-associated degradation of Ca_V_2.1 WT by RNF138. In the ER, proper folding of newly synthesized membrane proteins is catalyzed by molecular chaperones, as well as folding enzymes such as peptidyl-prolyl *cis/trans* isomerase (PPIase) [9, 16, 22]. The molecular nature of Ca_V_2.1 protein folding, however, is virtually unknown. In this study, we report the identification of a novel Ca_V_2.1 binding partner, peptidyl-prolyl *cis/trans* isomerase, NIMA-interacting 1 (Pin1). We provide multiple lines of evidence indicating that Pin1 interacts with specific Ca_V_2.1 protein domains harboring Pin1-binding motif, and thereby promotes Ca_V_2.1 degradation by RNF138. Importantly, our data support the idea that Pin1 plays an essential role in mediating defective proteostasis of EA2-causing mutants, as well as their dominant-negative effects on Ca_V_2.1 proteostasis.

## Materials and methods

### cDNA constructs

Human long carboxy-terminal α_1A_ subunit (Ca_V_2.1; AF004884) [35] was sublconed into pcDNA3.1 (Invitrogen, Carlsbad, CA, USA) or modified pcDNA3-Myc vectors as described previously [24]. The distal carboxy-terminal (dCT; amino acids 2204–2510) region of human Ca_V_2.1 long-isoform was subcloned into pcDNA3-Myc. Rat Pin1 (NM_001106701) was subcloned into pcDNA3-Myc, pcDNA3-Flag, or pcDNA3-HA. Rat RNF138 (long isoform; XM_039097200) was subcloned into pcDNA3-Myc or pcDNA3-Flag. Other cDNAs employed in this study include human short carboxy-terminal α_1A_ subunit (Ca_V_2.1-Short; AF004883; kindly provided by Dr. Jörg Striessnig, University of Innsbruck) [85], rat α_1C_ subunit (Ca_V_1.2; M67515; kindly provided by Dr. Gerald Obermair, Medical University of Innsbruck) [62], bovine α_1B_ subunit (Ca_V_2.2; AF173882; kindly provided by Dr. Aaron Fox, University of Chicago) [10], human Ca^2+^ channel auxiliary α_2_δ and β_4a_ subunits (NM_000722 & U95020), bovine β_1b_ and β_2a_ (AF174415 & AF174417), and HA-tagged human lysine-less ubiquitin (Ub-K0; Addgene 11934, Watertown, MA, USA).

### Cell culture and DNA transfection

Human embryonic kidney (HEK) 293T, rat pheochromocytoma PC12, and mouse hippocampal HT-22 cells were grown in Dulbecco’s modified Eagle’s medium (DMEM) (GIBCO, Gaithersburg, MD, USA) supplemented with 2 mM glutamine, 10% heat-inactivated fetal bovine serum (Hyclone, South Longan, UT, USA), 100 units/ml penicillin, and 50 µg/ml streptomycin, and were maintained at 37 °C in a humidified incubator with 95% air and 5% CO_2_. Transient transfection for HEK293T cells was performed by using the Lipofectamine 2000 (LF2000) reagent (Invitrogen). In brief, cells were plated onto 6- or 12-well plates 24 h before transfection. cDNA constructs were incubated with LF2000 reagent for 20 min at room temperature, and DNA-Lipofectamine diluted in Opti-MEM (Invitrogen) was applied to culture wells. After 6-hr incubation at 37ºC, the medium was changed. Transient transfection for HT-22 cells was performed by using the NTRII transfection reagent (T-Pro biotech, New Taipei city, Taiwan). Briefly, cDNA constructs were incubated with the NTRII reagent for 20 min at room temperature, and DNA-NTRII diluted in Opti-MEM was applied to culture wells. After 3-hr incubation at 37ºC, the medium was changed to Neurobasal medium supplemented with 2% B27, 2 mM GlutaMax I (Invitrogen), and 100 units/ml penicillin. Transfected HEK293T and HT-22 cells were maintained in a humidified 5% CO_2_ incubator at 37˚C for 24–48 h before being used for biochemical experiments. Where indicated, transfected HEK293T cells were incubated in the presence of 0.1% dimethyl sulfoxide (DMSO) (Sigma, St. Louis, MO, USA), 10 µM MG132 (Sigma), 25 or 50 µM all-*trans* retinoic acid (ATRA) (Sigma), or 10 µM PiB (Sigma) for 24 h.

### Rat brain lysates, subcellular fractionation, and cortical neuron culture

Procedures for dissecting forebrains from adult Sprague-Dawley rats, as well as retrieving embryos from pregnant rats, are in accordance with the Guidelines for the Care and Use of Mammals in Neuroscience and Behavioral Research (National Research Council 2003) and approved by the Institutional Animal Care and Use Committee (IACUC) of College of Medicine, National Taiwan University. Rat forebrains were homogenized with a motor driven glass-Teflon homogenizer in ice-cold dissociation buffer [(in mM) 320 sucrose, 1 MgCl_2,_ 0.5 CaCl_2,_ 1 NaHCO_3,_ 1 PMSF and 1 mg/l leupeptin)], and the cell debris was removed by centrifugation at 1,400x*g* for 10 min. The supernatant was saved, and the pellet was resuspended by homogenization in ice-cold dissociation buffer and pelleted again. The remaining pellet was discarded and the combined supernatants were pelleted (13,800x*g* for 10 min) again. The final pellet was resuspended in buffer A supplemented with 1% Triton X-100 and protease inhibitor cocktail (Roche, Basel, Switzerland).

For subcellular fractionation, rat forebrains were homogenized in buffer H1 [(in mM) 320 sucrose, 1 NaHCO_3_, 0.5 CaCl_2_, 0.1 PMSF] containing protease inhibitor cocktail, and centrifuged at 1,400x*g* to remove nuclei and other large debris (P1). The S1 fraction was subject to centrifugation at 13,800x*g* to obtain the crude synaptosome fraction (P2). The pellet was resuspended in buffer H2 [(in mM) 0.32 M sucrose and 1 mM NaHCO_3_)] and layered onto the top of the discontinuous sucrose density gradient by using 0.85, 1.0, and 1.2 M sucrose layers. The gradient was centrifuged at 65,000x*g* for 2 h in a Beckman Instruments SW-28 rotor and the synaptosomal fraction (SPM) was recovered from the 1.0-1.2 M sucrose interface. The synaptosomal fraction was extracted in ice-cold 0.5% Triton X-100/50 mM Tris-HCl (pH 7.9) for 15 min and centrifuged at 32,000x*g* for 45 min to obtain the PSD I pellet. The pellet was resuspended and further extracted a second time with 0.5% Triton X-100/50 mM Tris-HCl (pH 7.9), followed by centrifugation at 200,000x*g* for 45 min to obtain the PSD II pellet. Protein concentration was determined by the BCA protein assay kit (Thermo Fisher Scientific, Waltham, MA, USA). For immunoblotting, 40 µg (H, S1, P2, and SPM) or 20 µg (SPM, PSD I, and PSD II) of proteins were employed.

Dissociated cortical cultures were prepared by following a published protocol [7] with minor modification. In brief, the forebrains of embryonic day 18 (E18) rat embryos were removed and placed in the Hank’s balanced salt solution that contains 10 mM HEPES (pH 7.4) and 1 mM sodium pyruvate. The cortex was dissected out and dissociated by incubation with 0.25% trypsin solution. The dissociated cells were plated on coverslips at a density of about 160 and 800 cells/mm^2^ for immunofluorescence and immunoblotting, respectively. Coverslips were pre-coated with poly-D-lysine (1 mg/ml) (Sigma) and laminin (15 µg/ml) (Sigma). Cultures were maintained in the Neurobasal media supplemented with B27 (2%) and GlutaMax I (0.5 mM).

### Glutathione S-transferase (GST) pull-down assays

GST fusion proteins were produced and purified by following the manufacturer’s instruction (Stratagene, Agilent Technology, Santa Clara, CA, USA). Briefly, the cDNA fragments for human long-isoform Ca_V_2.1 amino-terminal region (Ca_V_2.1-N-ter; amino acids 1–99), cytoplasmic loop segment connecting transmembrane domains I and II (I-II loop) (Ca_V_2.1-I-II; amino acids 361–487), II-III loop (Ca_V_2.1-II-III; amino acids 715–1245), III-IV loop (Ca_V_2.1-III-IV; amino acids 1512–1567), proximal carboxy-terminal region (Ca_V_2.1-pC-ter; amino acids 1816–2203), and distal carboxy-terminal region (Ca_V_2.1-C-ter; amino acids 2204–2510), as well as human Pin1 (Addgene 19027), were subcloned into the pGEX vector (GE Healthcare Biosciences, Piscataway, NJ, USA) and expressed in the *E. coli* strain BL21. Bacterial cultures were grown at 30 °C, induced with 0.1 mM isopropyl-β-D-thiogalactopyranoside, and then harvested by centrifugation at 8,000x*g* for 10 min at 4ºC. Cell pellets were resuspended in the B-PER reagent (Pierce, Thermo Fisher Scientific) containing 1 mM phenylmethylsulfonyl fluoride (PMSF) and protease inhibitor cocktail. The lysates were clarified by centrifugation at 15,000x*g* for 15 min, and glutathione-agarose beads (Sigma) were used to bind the GST fusion proteins from the supernatant. GST protein-coated beads (4–8 µg) were incubated with pre-cleared cell lysates at 4ºC overnight. The bead-protein complexes were then washed with buffer A [(in mM) 100 NaCl, 4 KCl, 2.5 EDTA, 20 NaHCO_3_, 20 Tris-HCl, pH 7.5, plus 1 PMSF, 1 Na_3_VO_4_, 1 NaF, 1 β-glycerophosphate] (with and without 1% Triton X-100), and the proteins were eluted by boiling for 5 min in the Laemmli sample buffer.

### Immunoprecipitation

HEK293T cells and rat brain lysates were used for immunoprecipitation. Transfected HEK293T cells were incubated in the presence of 10 µM MG132 for 24 h, and then solubilized in ice-cold lysis buffer containing the protease inhibitor cocktail. Insolubilized materials were removed by centrifugation. Solubilized HEK293T cell lysates and rat brain homogenates were pre-cleared with protein A/G sepharose beads (GE Healthcare Biosciences, Piscataway, NJ, USA) for 1 h at 4 °C, and then incubated for 16 h at 4 °C with protein A/G sepharose beads pre-coated with appropriate antibodies. Beads were gently spun down and washed twice in a wash buffer [(in mM) 100 NaCl, 4 KCl, 2.5 EDTA, 20 NaHCO_3_, 20 Tris-HCl, pH 7.5] supplemented with 0.1% Triton X-100, and then twice with the wash buffer. The immune complexes were eluted from the beads by heating at 70 °C for 5 min in the Laemmli sample buffer.

### Immunoblotting

Transfected HEK293T cells were washed twice with ice-cold PBS [(in mM) 136 NaCl, 2.5 KCl, 1.5 KH_2_PO_4,_ 6.5 Na_2_HPO_4_, pH 7.4], centrifuged, and resuspended in a lysis buffer [(in mM) 150 NaCl, 5 EDTA, 50 Tris-HCl pH 7.6, 1% Triton X-100) containing protease inhibitor cocktail. After adding the Laemmli sample buffer to the HEK293T cells or rat brain lysates, samples were sonicated on ice (three times for five seconds each) and heated at 70 °C for 5 min. Samples were then separated by 7.5–10% SDS-PAGE, electrophoretically transferred to nitrocellulose membranes, and detected using rabbit anti-β-actin (1:5000; Bethyl Laboratories, Montgomery, TX, USA), rabbit anti-human Ca_V_2.1-CT [1:2500; the carboxy-terminal region (amino acids 2369–2473) of human Ca_V_2.1 long-isoform], rabbit anti-human Ca_V_2.1-(II-III) [1:2500; the linker region (amino acids 888–904) between transmembrane domains II and III of human Ca_V_2.1], rabbit anti-rat Ca_V_2.1 (1:200; Alomone, Jerusalem, Israel), rabbit anti-Flag (1:5000; Sigma), rabbit anti-GAPDH (1:5000; Bethyl), mouse anti-GST tag (1:5000; EnoGene, New York, NY, USA), rat anti-HA (1:5000; Roche, Basel, Switzerland), mouse anti-MPM2 (1:500; Abcam, Cambridge, UK), mouse anti-Myc (1:5000; clone 9E10), rabbit anti-Pin1 (1:1000; GeneTex, Hsinchu, Taiwan), mouse anti-PSD95 (1:5000; UC Davis/NIH NeuroMab Facility, Davis, CA, USA), rabbit anti-RNF138 (1:200; Abcam), mouse anti-synaptophysin (1:5000; clone 6D5AC4), rabbit anti-α-tubulin (1:5000; Bethyl), or mouse anti-ubiquitin (FK2) (1:1000; Enzo Life Sciences, Farmingdale, NY, USA) antibodies. Blots were then exposed to horseradish peroxidase-conjugated goat anti-mouse IgG (1:5000; Jackson ImmunoResearch), goat anti-rabbit IgG (1:5000; Jackson ImmunoResearch, West Grove, PA, USA), or goat anti-rat IgG (1:5000; Santa Cruz, Dallas, TX, USA), and revealed by an enhanced chemiluminescence detection system (Thermo Fisher Scientific). Chemiluminescent signals from immunoblots were acquired with the UVP AutoChemi image system (Ultra-Violet Products, Upland, CA, USA).

Densitometric scans of immunoblots were quantified by using ImageJ (National Institute of Health, Bethesda, MD, USA). For a given experiment on a specific scientific theme, all control and experimental groups were always performed in triplicates and subject to electrophoretical separation on the same gel, as well as densitometric analyses on the same immunoblot. For each sample on the same immunoblot, protein density was first standardized as the ratio to the signal of the cognate loading control. The average value of the standardized protein densities of the three independent control samples was then employed as the basis for the normalization of standardized protein densities for the control group, as well as for all the corresponding experimental groups. Normalized data from additional experiments on the same scientific theme were later pooled together for comprehensive statistical analyses. Numerical data were compiled from at least three independent experiments.

### Immunofluorescence

Coverslips containing cortical neurons were rinsed in PBS and then fixed with methanol (at 4 °C) or 4% paraformaldehyde (at room temperature) in PBS for 20 min. Cells were then permeabilized and blocked with a blocking buffer (5% normal goat serum in 20 mM phosphate buffer, pH 7.4, 0.1% (v/v) Triton X-100, and 0.45 M NaCl) for 60 min at 4 °C. Appropriate dilutions of primary antibodies were applied in the blocking buffer overnight at 4 °C. Immunoreactivities were visualized with goat-anti-mouse antibodies conjugated to Alexa568 (1:200; Invitrogen) or with goat anti-rabbit antibodies conjugated to Alexa488 (1:200; Invitrogen).

Fluorescence images were viewed and acquired with a TCS SP8 laser-scanning confocal microscope (Leica, Wetzlar, Germany) with the LAS AF software. Imaging data were further processed and analyzed with Photoshop CS6 (Adobe, San Jose, CA, USA) and ImageJ. To quantify the number of protein clusters per fixed length of neurites in cultured cortical neurons, built-in “set scale” and “freehand tool” functions of ImageJ were employed to trace multiple 100-µm neurite segments, followed by counting the number of Ca_V_2.1/Pin1/synaptophysin/PSD95 puncta within each 100-µm neurite segment. Colocalization of Ca_V_2.1/Pin1 (green punctate pixels) with synaptophysin/PSD-95 (red punctate pixels) puncta was quantified as the fraction of puncta associated with both green and red punctate pixels within 100-µm neurite segments.

### RNA interference

Lentivirus-based short hairpin RNA (shRNA) constructs (subcloned into the pLKO.1 vector) targeting specific human Pin1 (shPin1-1: 5’-CCAGAAGATCAAGTCGGGAGA-3’; shPin1-2: 5’-GCCATTTGAAGACGCCTCGTT-3’) sequences were purchased from National RNAi Core Facility, Taiwan. The shRNA for GFP (shGFP: 5’-GACCACCCTGACCTACGGCGT-3’) was used as a control. Recombinant lentivirus was generated by transfecting HEK293T cells with the packaging plasmid pCMV-ΔR8.91, the envelope plasmid pMD.G, and shRNA expressing constructs. The virus-containing supernatant was harvested and concentrated by ultracentrifugation to yield the viral stock, which in turn was supplemented with 8 µg/ml of polybrene for infection of HEK293T, PC12, or cortical cells. The infected cells were selected by 5 µg/ml of puromycin and subsequently transfected with the cDNA for Ca_V_2.1. For suppressing endogenous Pin1 in cultured cortical neurons, DIV9 neurons were infected with shRNA. After puromycin selection, infected DIV12 neurons were subject to immunoblotting analyses.

### Cycloheximide chase

24 h post-transfection, HEK293T cells were treated with the protein synthesis inhibitor cycloheximide (CHX) (100 µg/ml) (Sigma) for 0–6 h, followed by immunoblotting. Quantitative analyses of protein degradation time course under various coexpression conditions were implemented by standardizing Ca_V_2.1 protein densities in response to different CHX treatment durations as the ratio of Ca_V_2.1 signal to the cognate tubulin signal, followed by normalization with respect to the corresponding vector control at 0 h. The logarithmic value of normalized attenuation of Ca_V_2.1 protein density was in turn plotted against a linear scale of CHX treatment duration (the semi-logarithmic plot). Ca_V_2.1 protein half-life value was then derived from single linear-regression analysis of the semi-logarithmic plot. For a given experimental condition with multiple repeats, individual Ca_V_2.1 protein half-life value was determined from each single trial, followed by data pooling and statistical analyses.

### Protein ubiquitination analyses

Transfected cells were treated with 10 µM MG132 at 37 °C for 24 h, and solubilized in the lysis buffer supplemented with 2.5 mg/ml N-ethylmaleimide to inactivate deubiquitinating enzymes, followed by immunoprecipitation with the anti-Myc antibody. To facilitate the visualization of ubiquitination signals associated with proteins of high molecular weight, we employed a 7% SDS-PAGE protocol involving a gel running time over 2 h.

### Cell surface biotinylation

Transfected cells were washed extensively with D-PBS (Sigma) supplemented with 0.5 mM CaCl_2_, 2 mM MgCl_2_, followed by incubation in 1 mg/ml sulfo-NHS-LC-biotin (Thermo Fisher Scientific) in D-PBS at 4 °C for 1 h with gentle rocking. Biotinylation was terminated by removing the biotin reagents and rinsing three times with 100 mM glycine in PBS, followed by once in TBS buffer [(in mM) 20 Tris-HCl, 150 NaCl, pH 7.4]. Cells were solubilized in a lysis buffer [(in mM) 150 NaCl, 50 Tris-HCl, 1% Triton X-100, 5 EDTA, 1 PMSF, pH 7.6] supplemented with the protease inhibitor cocktail. Insolubilized materials were removed by centrifugation. Solubilized cell lysates were incubated overnight at 4 °C with streptavidin-agarose beads (Thermo Fisher Scientific). Beads were washed once in the lysis buffer, followed by twice in a high-salt buffer [(in mM) 500 NaCl, 5 EDTA, 50 Tris-HCl, pH 7.6, 0.1% Triton X-100] and once in a low-salt buffer [(in mM) 2 EDTA, 10 Tris-HCl, pH 7.6, 0.1% Triton X-100]. The biotin-streptavidin complexes were eluted from the beads by heating at 70 °C for 5 min in the Laemmli sample buffer.

### Electrophysiological recordings

Oocytes from adult female *Xenopus* laevis (Xenopus 1 Corp., Dexter, MI, USA) were employed for functional studies of human Ca_V_2.1 currents as described previously [38]. *Xenopus* frogs were anesthetized by immersion in ice water containing Tricaine (1.5 g/liter) in conformity with the animal protocol approved by IACUC of National Taiwan University. Ovarian follicles were removed, cut into small pieces, and incubated in the ND96 solution [(in mM) 96 NaCl, 2 KCl, 1.8 MgCl_2_, 1.8 CaCl_2_, and 5 HEPES, pH 7.2]. To remove the follicular membrane, *Xenopus* oocytes were incubated in the Ca^2+^-free ND96 solution containing collagenase (2 mg/ml) on an orbital shaker (~ 200 rpm) for about 60–90 min at room temperature. After several washes with collagenase-free, Ca^2+^-free ND96, oocytes were transferred to ND96. For in vitro transcription, cDNAs were linearized with appropriated restriction enzymes. Capped cRNAs were transcribed from linearized cDNA templates with the mMessage mMachine T7 kit (Ambion, Thermo Fisher Scientific). Stage V-VI *Xenopus* oocytes were selected for cRNA injection. cRNAs for α_1A_, α_2_δ, and β_4_ were mixed in a molar ratio of 1:2:2, and the final injection amount (total volume: 41.4 nl) for α_1A_, α_2_, and β_4_ cRNAs were ~ 3.8 ng, ~ 3.8 ng, and ~ 1.9 ng per oocyte, respectively. Injected oocytes were stored at 16 °C in ND96.

2–3 days after cRNA injection, *Xenopus* oocytes were transferred into a recording bath (~ 200 µl) containing (in mM) 40 Ba(OH)_2_, 50 NaOH, 2 CsOH, and 5 HEPES (pH 7.4 with methanesulfonic acid). An agarose bridge was used to connect the bath solution to a ground chamber (containing 3 M KCl), into which two ground electrodes were inserted. Borosilicate electrodes (0.1–1 MΩ) used in voltage recording and current injection were filled with 3 M KCl. Ba^2+^ currents through Ca_V_2.1 channels were acquired using the conventional two-electrode voltage clamp technique with the Warner OC-725 C oocyte clamp (Harvard Apparatus, Holliston, MA, USA). The holding potential was set at -90 mV and passive membrane properties were compensated using the -P/4 leak subtraction method provided by the pCLAMP software (Molecular Devices, San Jose, CA, USA). Data were sampled at 10 kHz and filtered at 1 kHz. All recordings were performed at room temperature (20–22 °C). To avoid potential biases imposed by variations in Ca_V_2.1 channel expression amongst different oocytes, for the same batch of oocytes on a given day of experimentation, the average value of the peak Ba^2+^ current amplitudes of Ca_V_2.1 channels under each coexpression condition was normalized with respect to that of the Ca_V_2.1 control. Normalized data from different batches of *Xenopus* oocytes on different dates were later pooled together for comprehensive analyses.

### qPCR and RT-PCR

RNA was extracted from HEK293T cells or cortical neurons with Trizol (Sigma), followed by phenol/chloroform separation. To eliminate potential plasmid DNA contamination in the RNA prepared from HEK293T cells transfected with Ca_V_2.1, we performed DNase I (Promega, Madison, WI, USA) treatment as a control experiment. RNA was reverse transcribed into cDNA using High-Capacity cDNA Reverse Transcription Kit with RNase Inhibitor (Thermo Fisher). The reaction mixture was incubated at 25 °C and 37 °C for 10 min and 120 min, respectively, followed by heating to 85 °C for 5 min.

For qPCR analyses in HEK293T cells, reverse transcription products were diluted with nuclease-free water and amplified in 25-µl reactions using SYBR Green PCR Master Mix (Thermo Fisher) and 500 nM forward and reverse primers. The specific primers used for qPCR reactions are: Ca_V_2.1 *forward* (5’- GAGGGTCCGGAGGACAAG-3’) and *reverse* (5’- TTCTCTTTCCTCCTCCGATG-3’); and GAPDH *forward* (5’-CAAGGTCATCCATGACAACTTTG-3’), and *reverse* (5’-GTCCACCACCCTGTTGCTGTAG-3’). The ABI QuantStudio 5 system (Thermo Fisher) was employed for signal detection. Relative mRNA amounts were calculated using the comparative threshold cycle CT (ΔΔC_T_) method. Briefly, for each experiment the average C_T_ values for Ca_V_2.1 and the endogenous control GAPDH were first calculated, and the ΔC_T_ value was generated by subtracting the mean GAPDH C_T_ value from the corresponding mean Ca_V_2.1 C_T_ value. The ΔΔC_T_ value was then determined by subtracting the ΔC_T_ value of the vector control from the ΔC_T_ of each of the corresponding Pin1 coexpression groups. Finally, the relative gene expression was expressed as 2^–ΔΔCT^.

For RT-PCR analyses in HEK293T cells and cultured neurons, PCR amplification with *Taq* polymerase (Bioman, New Taipei City, Taiwan) was carried out as follows: 95 °C for 5 min; non-saturating 20 (HEK293T cells) or 25 (cortical neurons) cycles at 95 °C for 30 s, 55 °C for 30 s, and 72 °C for 1 min; and a final extension at 72 °C for 7 min. The specific primers used for PCR reactions are: (HEK293T cells) Ca_V_2.1 *forward* (5’-TGGAATCAGCGTGTTACGAG-3’) and *reverse* (5’-GCGGACAGAGGACTCACTTC-3’); (cortical neurons) Ca_V_2.1 *forward* (5’-TGGAATCAGCGTGTTACGAG-3’), and *reverse* (5’-GCAGACAGGGGACTCACTTC-3’); and GAPDH *forward* (5’-CAAGGTCATCCATGACAACTTTG-3’), and *reverse* (5’-GTCCACCACCCTGTTGCTGTAG-3’). The predicted sizes of the amplified PCR fragment for Ca_V_2.1 and GAPDH are about 500 and 496 bp, respectively. RT-PCR products were resolved by electrophoresis in 1.7% agarose gels, followed by quantification with ImageJ.

### Statistical analyses

Statistical analyses were performed with Origin 7.0 (OriginLab, Northampton, MA, USA) and Prism 6.0 (GraphPad Software, Boston, MA, USA). The significance of the difference between two means was tested using Student’s *t*-test. Means from multiple groups were compared using one-way ANOVA, followed by *post-hoc* analyses with Bonferroni test. All numerical values are presented as mean ± SD.

## Results

### Pin1 is a novel binding partner of CaV2.1 in neurons

**Fig. 1:**
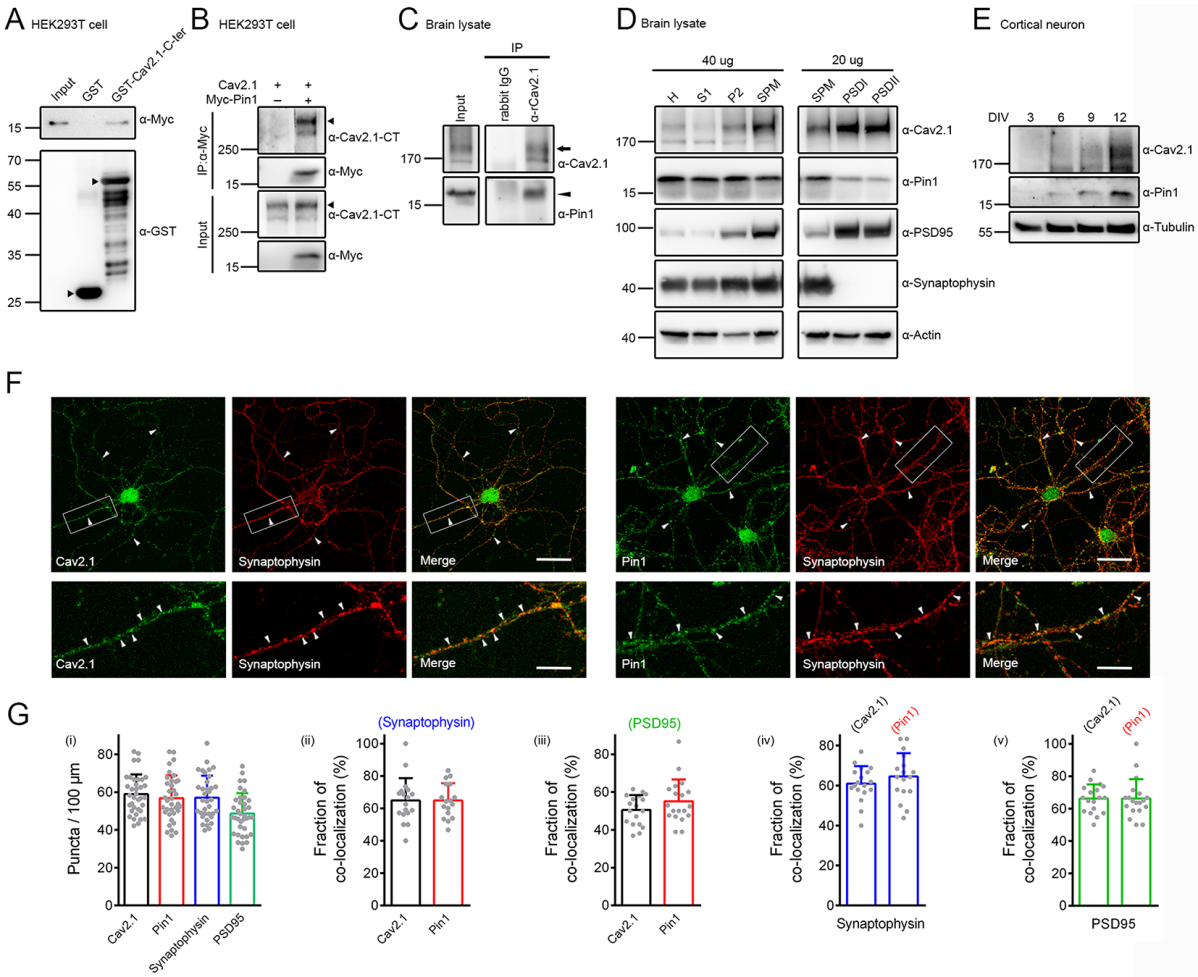
Interaction and colocalization of Ca_V_2.1 and Pin1. **A** Representative immunoblots showing the association of Myc-tagged rat Pin1 (*Myc-Pin1*) with the GST fusion protein comprising human Ca_V_2.1 distal carboxy-terminal fragment (*GST-Ca*_*V*_*2.1-C-ter*), but not the GST protein per se. Lysates from HEK293T cells overexpressing Myc-Pin1 were subject to GST pull-down assay, followed by immunoblotting with the anti-Myc antibody (*α-Myc*). GST and GST-Ca_V_2.1 fusion proteins were detected with the anti-GST antibody (*α-GST*). *Input* represents ~ 1% of total cell lysate volume. Arrowheads denote the location of GST or GST-Ca_V_2.1 fusion protein bands. Molecular weight markers (in kDa) are labeled to the left. Arrowheads denote the corresponding GST fragments. **B** Representative immunoblots demonstrating the coimmunoprecipitation of Myc-Pin1 with the human long carboxy-terminal Ca_V_2.1 in HEK293T cells. Coexpression with the Myc vector was used as the control. HEK293T cell lysates were immunoprecipitated (*IP*) with α-Myc, followed by immunoblotting with α-Myc or the anti-human Ca_V_2.1 carboxy-terminal antibody (*α-Ca*_*V*_*2.1-CT*). The apparent molecular weight of human Ca_V_2.1 long-isoform is ~ 280–290 kDa (*arrowhead*). Corresponding expression level of human Ca_V_2.1 and Myc-Pin1 in the lysates is shown in the *Input* lane, which represents ~ 10% of the total protein used for immunoprecipitation. **C** Representative immunoblots displaying the interaction of endogenous Ca_V_2.1 (*arrow*) and Pin1 (*arrowhead*) in the rat brain. Endogenous rat Ca_V_2.1 in the brain comprises a major isoform with an apparent molecular weight ~ 190 kDa, as well as a minor isoform of ~ 220 kDa. Rat forebrain lysates were immunoprecipitated with the anti-rat Ca_V_2.1 antibody (*α-Ca*_*V*_ *2.1*) or the rabbit IgG. **D** Representative immunoblots depicting the colocalization of endogenous Ca_V_2.1 and Pin1 at presynaptic and postsynaptic compartments in the rat brain. Following subcellular fractionation, rat brain lysates were separated into homogenate (*H*), soluble (*S1*), crude membrane (*P2*), synaptosomal (*SPM*), and two postsynaptic density (*PSD I*,* PSD II*) fractions, with synaptophysin and PSD95 serving as the presynaptic and the postsynaptic markers, respectively. The labels 40 µg and 20 µg denote the amount of total protein loaded in each lane. **E** Representative immunoblots illustrating corresponding protein level of endogenous Ca_V_2.1 and Pin1 in cultured rat cortical neurons with the indicated days in vitro (*DIV*). **F** Representative confocal images of endogenous Ca_V_2.1 (*left panels*) and Pin1 (*right panels*) immunofluorescent signals in rat DIV10 cortical neurons, showing colocalization of Ca_V_2.1 or Pin1 (*green*) with synaptophysin-puncta (*red*) along neurites, as denoted by arrowheads and further highlighted by yellow puncta in the merge images. The boxed regions in the images shown in the upper rows are magnified for detailed inspection in the corresponding lower rows. Scale bars, 50 μm (*upper rows*) and 12.5 μm (*lower rows*). See Supplementary Figure S1 for additional confocal images exemplifying the colocalization of Ca_V_2.1/Pin1 with MAP2, tau, or PSD-95. **G** Quantification of puncta density (*Puncta/100 µm*) and puncta colocalization ratio (*Fraction of colocalization*) in rat DIV10 cortical neurons. (*i*) Puncta densities per 100-*µ*m neurite: 57.17 ± 9.32 (Ca_V_2.1), 59.39 ± 12.50 (Pin1), 53.19 ± 10.53 (synaptophysin), and 49.69 ± 12.09 (PSD95). (*ii-iii*) The fraction of Ca_V_2.1 and Pin1 puncta colocalized with synaptophysin is ~ 64.80 ± 13.80% and 64.78 ± 10.71%, respectively. The fraction of Ca_V_2.1 and Pin1 puncta colocalized with PSD95 is ~ 50.48 ± 7.93% and 55.08 ± 11.61%, respectively. (*iv-v*) The fraction of synaptophysin puncta colocalized with Ca_V_2.1 and Pin1 is ~ 60.97 ± 8.77% and 64.51 ± 11.65%, respectively. The fraction of PSD95 puncta colocalized with Ca_V_2.1 and Pin1 is ~ 66.12 ± 8.79% and 66.15 ± 12.08%, respectively. The data were compiled from 17–25 individual neurites associated with 3–6 different cortical neurons

Using yeast two-hybrid screening of a rat brain cDNA library with the distal carboxy-terminal region of a long carboxy-terminal isoform (AF004884; encoded by exon 47; +47) [77] of human Ca_V_2.1 subunit, we identified Pin1 as a potential binding partner of Ca_V_2.1 [24]. In vitro interaction between Ca_V_2.1 and Pin1 was further verified with GST pull-down and immunoprecipitation assays using the HEK293T heterologous expression system (Fig. 1A-B). We also demonstrated in Fig. 1C that, in the rat forebrain, endogenous Pin1 was coimmunoprecipitated with Ca_V_2.1, consistent with the idea that Ca_V_2.1 and Pin1 may coexist in the same protein complex in native neurons.

In the brain, Ca_V_2.1 is abundantly present in the synaptic region, localized at both presynaptic axon terminals and postsynaptic dendritic spines [45, 48, 49, 73, 87]. Pin1 is also known to be present at synapses [5, 88]. To investigate whether Ca_V_2.1 may interact with Pin1 at neuronal synapses, we then studied their subcellular localization in neurons. As illustrated in Fig. 1D, sucrose gradient centrifugation analyses of rat forebrain homogenates revealed that both Ca_V_2.1 and Pin1 were enriched in the synaptosomal (SPM) fraction, as well as in the SPM sub-fractions postsynaptic density (PSD) I and PSD II, indicating that Ca_V_2.1 and Pin1 may colocalize at both presynaptic and postsynaptic compartments.

In addition, cultured cortical neurons prepared from rat embryos were employed for morphological analyses of Ca_V_2.1 and Pin1 localization. Since endogenous Ca_V_2.1 and Pin1 were present in nine days in vitro (DIV9) neurons (Fig. 1E), DIV10 neurons were chosen for confocal microscopic studies. Supplementary Figure S1 shows that immunofluorescent signals of Ca_V_2.1 and Pin1 were readily detected in both dendrites (MAP2-positive neurites) and axons (tau-positive neurites). Moreover, in agreement with previous demonstration of distinct punctate immunostaining pattern of synaptic Ca_V_2.1 in brain sections [49, 73, 87], Ca_V_2.1 and Pin1 displayed comparable punctate colocalization with the presynaptic marker synaptophysin and the postsynaptic marker PSD-95 in cultured cortical neurons (Fig. 1F-G; Suppl. Fig. S1). Altogether, these observations support the association and colocalization of endogenous Ca_V_2.1 and Pin1 in neurons.

### Pin1 promotes CaV2.1 protein degradation

**Fig. 2:**
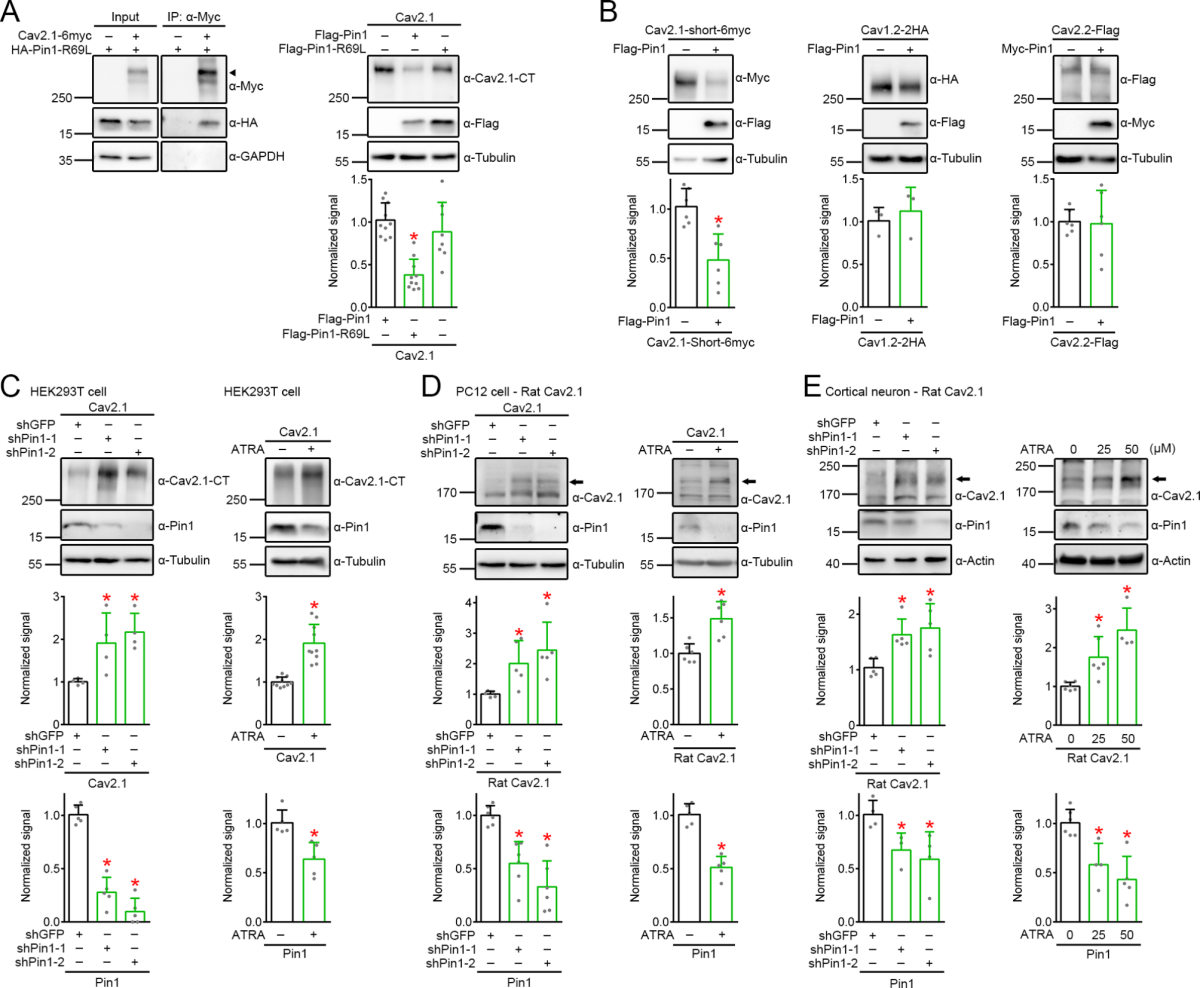
Reduction of Ca_V_2.1 protein expression by Pin1. **A** (*Left*) Representative immunoblots showing the coimmunoprecipitation of Myc-tagged human long carboxy-terminal Ca_V_2.1 (*Ca*_*V*_*2.1-6myc*) with HA-tagged Pin1 R69L mutant (*HA-Pin1-R69L*) in HEK293T cells. Cells transfected with Myc vector (−) were used as control. Arrowhead denotes the primary Ca_V_2.1 protein band. (*Right*) Representative immunoblots (*top*) and quantification (*bottom*) of the effect of coexpression (in the molar ratio 3:1) with Flag vector (−), Flag-tagged Pin1 (*Flag-Pin1*) or Flag-tagged Pin1-R69L mutant (*Flag-Pin1-R69L*) on Ca_V_2.1 protein level in HEK293T cells. Tubulin expression was chosen as the loading control. Total Ca_V_2.1 protein density was standardized as the ratio to the cognate tubulin signal, followed by normalization with respect to the corresponding vector control. Normalized Ca_V_2.1 protein levels (*n* = 9–10): vector, 1.02 ± 0.20; Pin1, 0.38 ± 0.18; Pin1-R69L, 0.89 ± 0.35. Asterisks denote significant difference from the vector control (*, *P* < 0.05). **B** (*Top panels*) Representative immunoblots demonstrating the effect of Pin1 coexpression on protein level of Myc-tagged human Ca_V_2.1 short-isoform (*Ca*_*V*_*2.1-Short-6myc*; ~260–270 kDa), HA-tagged rat Ca_V_1.2 (*Ca*_*V*_*1.2-HA*; ~260 kDa), or Flag-tagged bovine Ca_V_2.2 (*Ca*_*V*_*2.2-Flag*; ~255–280 kDa) in HEK293T cells. (*Bottom panels*) Quantification of relative Ca^2+^ channel protein level (*n* = 4–6). Ca_V_2.1-short: vector, 1.03 ± 0.18; Pin1, 0.48 ± 0.26. Ca_V_1.2: vector, 1.01 ± 0.16; Pin1, 1.12 ± 0.28. Ca_V_2.2: vector, 1.00 ± 0.14; Pin1, 0.98 ± 0.39. Asterisks denote significant difference from the vector control (*, *P* < 0.05). **C-E** (*Top panels*) Representative immunoblots displaying the effect of shRNA knockdown (*shPin1-1*,* shPin1-2*) (*left*) and ATRA-induced suppression (*right*) of endogenous Pin1 expression on protein level of overexpressed human Ca_V_2.1 in HEK293T cells (**C**), or endogenous rat Ca_V_2.1 in PC12 cells (**D**) and cultured cortical neurons (**E**). Cells were subject to treatment with 25 or 50 µM ATRA for 24 h. shGFP and DMSO were employed as infection and drug treatment controls, respectively. Tubulin and actin were used as loading controls. Arrows refer to the endogenous 190-kDa rat Ca_V_2.1. (*Middle panels*) Quantification of relative Ca^2+^ channel protein levels (*n* = 4–10). Total Ca_V_2.1 protein density was standardized as the ratio to the loading control, followed by normalization with respect to the corresponding shGFP or DMSO control. HEK293T cells: (*left*) shGFP (1.00 ± 0.07), shPin1-1 (1.91 ± 0.71), shPin1-2 (2.17 ± 0.44); (*right*) DMSO (1.00 ± 0.11), ATRA (1.91 ± 0.44). PC12 cells: (*left*) shGFP (1.00 ± 0.09), shPin1-1 (2.01 ± 0.75), shPin1-2 (2.44 ± 0.92); (*right*) DMSO (1.00 ± 0.13), ATRA (1.49 ± 0.24). Cortical neurons: (*left*) shGFP (1.04 ± 0.16), shPin1-1 (1.63 ± 0.28), shPin1-2 (1.75 ± 0.44); (*right*) DMSO (1.04 ± 0.16), 25 µM ATRA (1.63 ± 0.28), 50 µM ATRA (1.75 ± 0.44). (*Bottom panels*) Quantification of relative Pin1 protein level (*n* = 3–8). Asterisks denote significant difference from the cognate shGFP or DMSO control (*, *P* < 0.05)

To assess the biochemical significance of Ca_V_2.1-Pin1 interaction, we first generated a mutant construct of Pin1, Pin1-R69L, which was substantially deficient in its isomerase activity [72], but retained the interaction with the long carboxy-terminal isoform of Ca_V_2.1 (Fig. 2A). Upon heterologous expression in HEK293T cells, Pin1, but not the R69L mutant, significantly reduced Ca_V_2.1 protein level by about 50% (Fig. 2A; Suppl. Fig. S2). In addition to the long carboxy-terminal isoform, there is another splice variant of human Ca_V_2.1 (AF004883; Δ47; hereafter referred to as Ca_V_2.1-short), with a distal carboxy-terminal region shorter by about 244 amino acids [2, 51, 77]. Pin1 also down-regulated the protein expression of Ca_V_2.1-short, but not L-type (Ca_V_1.2) or N-type (Ca_V_2.2) Ca^2+^ channel subunits (Fig. 2B).

Conversely, shRNA knockdown of endogenous Pin1 expression substantially enhanced human Ca_V_2.1 protein level in HEK293T cells (Fig. 2C). A similar Pin1 knockdown approach significantly promoted endogenous rat Ca_V_2.1 protein expression in the adrenal medulla-derived pheochromocytoma cell line PC12 (Fig. 2D). Alternatively, we diminished endogenous Pin1 level by employing a well-known therapeutic drug for leukemia, all-*trans* retinoic acid (ATRA), which directly binds to and promotes degradation of Pin1 [86]. Figure 2C shows that 24 h-treatment with 50 µM ATRA resulted in about 40% decrease in endogenous Pin1 level in HEK293T cells, as well as about 90% increase in Ca_V_2.1 protein expression. Protein expression of endogenous Ca_V_2.1, but not Ca_V_1.2, in PC12 cells was also markedly boosted by ATRA treatment (Fig. 2D). Importantly, suppression of Pin1 expression in cultured rat cortical neurons, via either shRNA knockdown or ATRA treatment, notably increased endogenous Ca_V_2.1 protein level (Fig. 2E).

**Fig. 3:**
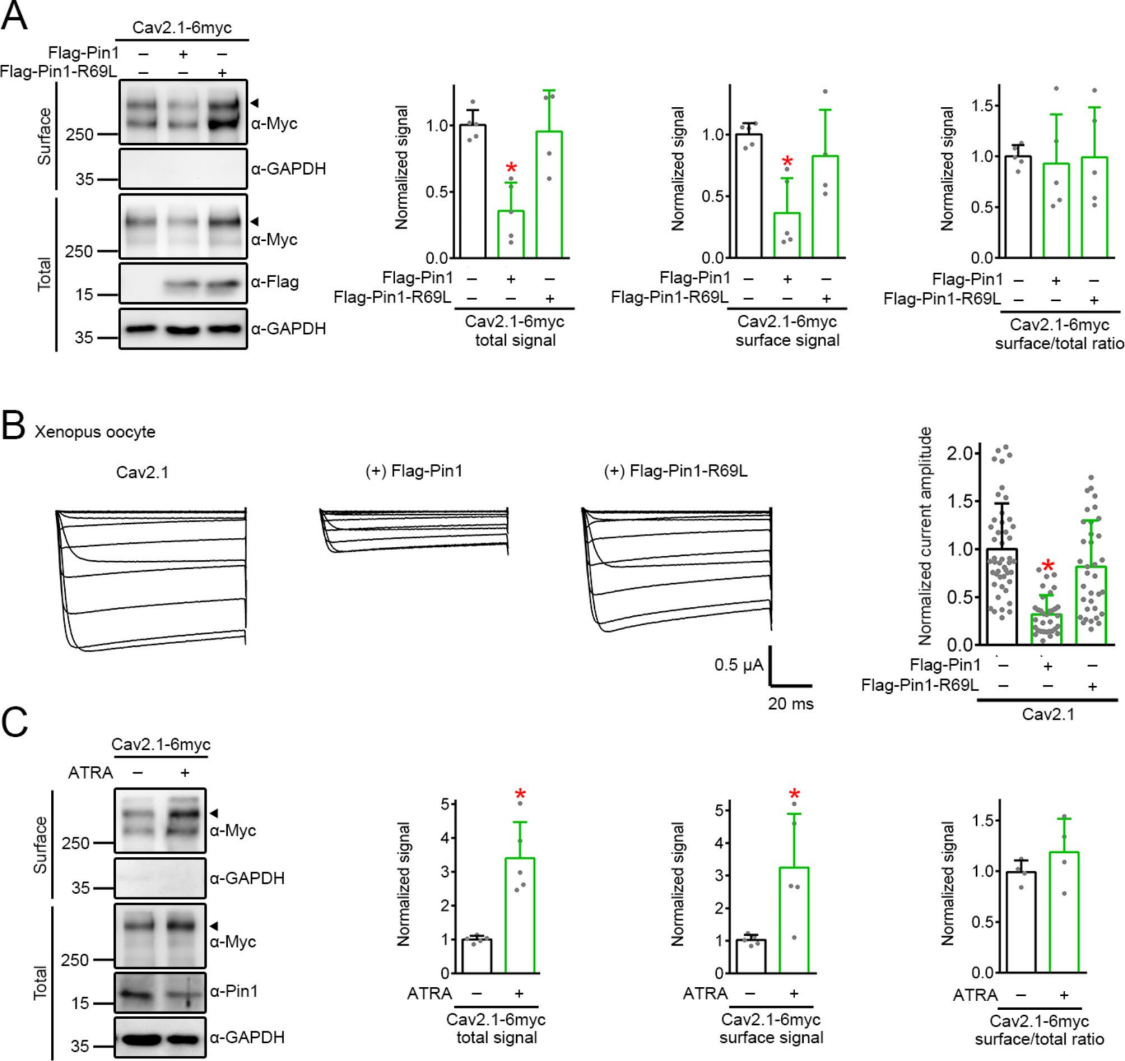
Attenuation of cell-surface and functional expression of Ca_V_2.1 by Pin1. **A** Surface biotinylation experiments in HEK293T cells coexpressing Ca_V_2.1-6myc with Flag vector (−), Flag-Pin1, or Flag-Pin1-R69L. (*Left*) Representative immunoblots showing cell-surface expression of human Ca_V_2.1. Lysates from biotinylated intact cells were either directly employed for immunoblotting analyses (*Total*), or subject to streptavidin pull-down prior to immunoblotting analyses (*Surface*). GAPDH was used as the loading control. Ca_V_2.1-6myc, α2δ, and β4a were coexpressed in the molar ratio 1:2:1. Arrowheads denote the primary Ca_V_2.1 protein band. (*Right panels*) Quantification of relative total protein level (*total signal*), surface protein level (*surface signal*), and membrane trafficking efficiency (*surface/total ratio*) of Ca_V_2.1 (*n* = 4–5). Total protein density was standardized as the ratio of total Ca_V_2.1 to GAPDH signals. Surface protein density was standardized as the ratio of surface Ca_V_2.1 to cognate total GAPDH signals. Membrane trafficking efficiency was calculated as the ratio of surface protein density to standardized total protein density. All data were normalized with respect to the corresponding vector control. Total Ca_V_2.1: vector, 1.00 ± 0.11; Pin1, 0.36 ± 0.21; Pin1-R69L, 0.96 ± 0.31. Surface Ca_V_2.1: vector, 1.00 ± 0.09; Pin1, 0.36 ± 0.28; Pin1-R69L, 0.83 ± 0.38. Ca_V_2.1 trafficking: vector, 1.00 ± 0.11; Pin1, 0.93 ± 0.48; Pin1-R69L, 0.99 ± 0.49. Asterisks denote significant difference from the vector control (*, *P* < 0.05). **B** Two-electrode voltage clamp experiments in *Xenopus* oocytes expressing Ca_V_2.1 in the absence or presence of Pin1, or Pin1-R69L. (*Left panels*) Representative Ba^2+^ current traces through human Ca_V_2.1 channel. From a holding potential of -90 mV, oocytes were subject to 70-ms test pulses ranging from − 80 to + 60 mV (in 10-mV increments). Oocytes coinjected with Ca_V_2.1 cRNA and water were used as the control. (*Right*) Quantification of relative peak Ba^2+^ current amplitude at + 20 mV (*n* = 33–47). Data were normalized with respect to the Ca_V_2.1-water coinjection control (−). Normalized Ca_V_2.1 current amplitude: control, 1.00 ± 0.48; Pin1, 0.32 ± 0.20; Pin1-R69L, 0.82 ± 0.48. Asterisks denote significant difference from the water control (*, *P* < 0.05). **C** (*Left*) Representative immunoblots comparing the effect of 24-hr treatment with DMSO (−) or 50 µM ATRA on cell-surface expression of human Ca_V_2.1 in HEK293T cells. Arrowheads denote the primary Ca_V_2.1 protein band. (*Right Panels*) Quantification of relative Ca_V_2.1 protein level and membrane trafficking efficiency (*n* = 5). Total Ca_V_2.1: DMSO, 1.00 ± 0.10; ATRA, 3.40 ± 1.07. Surface Ca_V_2.1: DMSO, 1.03 ± 0.16; ATRA, 3.25 ± 1.65. Ca_V_2.1 trafficking: DMSO, 1.00 ± 0.12; ATRA, 1.19 ± 0.33. Asterisks denote significant difference from the DMSO control (*, *P* < 0.05)

**Fig. 4:**
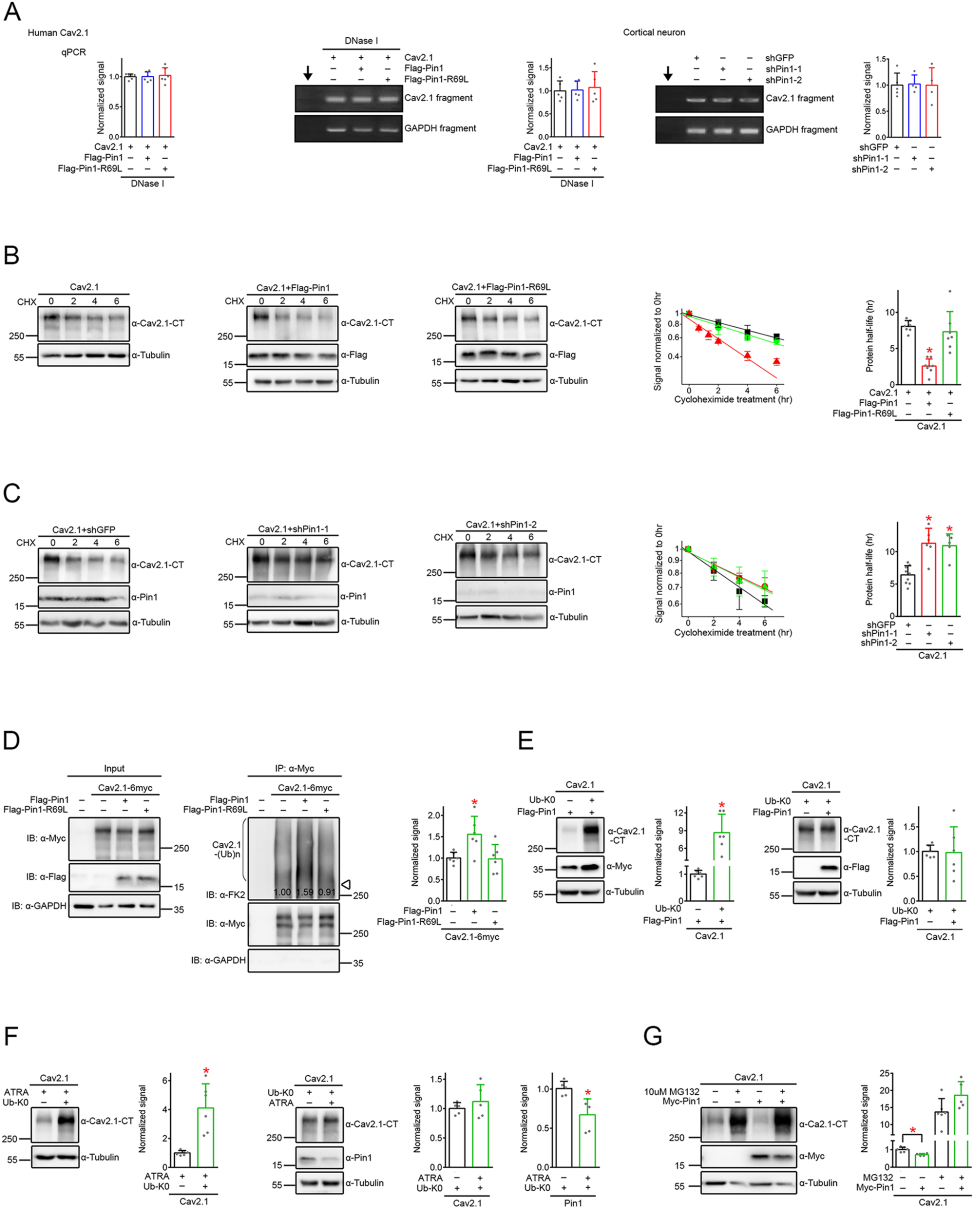
Disruption of Ca_V_2.1 protein stability by Pin1. **A** (*Left & central panels*) Lack of effect of Pin1 overexpression on human Ca_V_2.1 mRNA level. Ca_V_2.1 was overexpressed in HEK293T cells along with Flag vector, Flag-Pin1, or Flag-Pin1-R69L. (*Left panel*) RNA extracted from HEK293T cells was reverse transcribed into cDNA, followed by qPCR analyses (*n* = 6). (*Central panels*) RNA extracted from HEK293T cells was reverse transcribed into cDNA, followed by RT-PCR analyses (*n* = 5). To rule out potential contamination arising from the human Ca_V_2.1 plasmid in HEK293T cells, DNase I was applied prior to the reverse transcription reaction. (*Right panels*) Lack of effect of Pin1 knockdown on rat Ca_V_2.1 mRNA level. shRNA knockdown of endogenous Pin1 expression was performed in cultured rat cortical neurons. RNA extracted from neurons was reverse transcribed into cDNA, followed by RT-PCR analyses (*n* = 3–4). For quantification, Ca_V_2.1 signals were standardized as the ratio to cognate GAPDH signals, followed by normalization with respect to the corresponding vector or shGFP control. Arrows refer to PCR in the absence of cDNA templates. **B-C** (*Left Panels*) Representative immunoblots showing the effect of Pin1 or Pin1 R69L overexpression (**B**), as well as shRNA knockdown of endogenous Pin1 (**C**), on human Ca_V_2.1 protein stability in HEK293T cells. The time course of Ca_V_2.1 protein turnover was analyzed by applying cycloheximide (CHX) for the indicated durations. Coexpression with Flag vector (**B**), as well as ShGFP knockdown (**C**), was used as the control experiment. (*Right Panels*) Quantification of human Ca_V_2.1 protein turnover kinetics (*n* = 7–9). Ca_V_2.1 protein density was normalized with respect to the corresponding value for no CHX treatment (0 h), followed by transformation into semilogarithmic plot and single linear-regression analyses. Ca_V_2.1 protein half-life values (hr): (**B**) vector (*black*), 8.06 ± 0.81; Pin1 (*red*), 2.57 ± 1.00; Pin1-R69L (*green*), 7.32 ± 2.79; (**C**) shGFP (*black*), 6.41 ± 1.42; shPin1-1 (*red*), 11.29 ± 2.31; shPin1-2 (*green*), 10.93 ± 1.82. Asterisks denote significant difference from the vector/shGFP control (*, *P* < 0.05). The difference between the Ca_V_2.1 protein half-life values for “Flag vector” and “shGFP knockdown” is statistically insignificant (*P* > 0.05). **D** (*Left Panels*) Representative immunoblots comparing the effect of Flag vector, Flag-Pin1, or Flag-Pin1 R69L on human Ca_V_2.1 polyubiquitination in HEK293T cells. Lysates from cells overexpressing Ca_V_2.1-6myc were immunoprecipitated with α-Myc, followed by immunoblotting with the anti-ubiquitin antibody α-FK2. Ca_V_2.1 polyubiquitination [*Ca*_*V*_*2.1-(Ub)n*] by endogenous ubiquitin is visualized as high-molecular-weight protein smears. Normalized densitometric Ca_V_2.1 ubiquitination intensity is labeled on the immunoblot. Corresponding expression level of Ca_V_2.1, Pin1, and GAPDH in the lysates is shown in the *Input* lane. Open triangle refers to the expected location of the protein band corresponding to non-ubiquitinated Ca_V_2.1. (*Right*) Quantification of relative Ca_V_2.1 ubiquitination signal (*n* = 6). Ca_V_2.1 ubiquitination signals were normalized with respect to the Flag vector control: vector, 1.01 ± 0.13; Pin1, 1.56 ± 0.42; Pin1 R69L, 0.98 ± 0.33. Asterisk denotes significant difference from the vector control (*, *P* < 0.05). **E** (*Left Panels*) Representative immunoblots and quantification of the effect of the lysine-less ubiquitin mutant Ub-K0 on Ca_V_2.1 regulation by Pin1 in HEK293T cells. Normalized Ca_V_2.1 protein level (*n* = 6): Pin1 + vector, 1.01 ± 0.14; Pin1 + Ub-K0, 8.67 ± 3.12. (*Right Panels*) Representative immunoblots and quantification of the effect of Pin1 on Ca_V_2.1 regulation by Ub-K0 in HEK293T cells. Normalized Ca_V_2.1 protein level (*n* = 6): Ub-K0 + vector, 1.00 ± 0.13; Ub-K0 + Pin1, 0.98 ± 0.52. Asterisks denotes significant difference (*, *P* < 0.05). **F** (*Left Panels*) Representative immunoblots and quantification of the effect of Ub-K0 on Ca_V_2.1 regulation by 50 µM ATRA in HEK293T cells. Normalized Ca_V_2.1 protein level (*n* = 6): ATRA + vector, 1.01 ± 0.17; ATRA + Ub-K0, 4.12 ± 1.67. (*Right Panels*) Representative immunoblots and quantification of the effect of ATRA treatment on Ca_V_2.1 regulation by Ub-K0 in HEK293T cells. Normalized Ca_V_2.1 protein level (*n* = 5): Ub-K0 + DMSO, 1.00 ± 0.10; Ub-K0 + ATRA, 1.12 ± 0.29. Normalized Pin1 protein level (*n* = 5): Ub-K0 + DMSO, 1.01 ± 0.09; Ub-K0 + ATRA, 0.67 ± 0.20. Asterisks denotes significant difference (*, *P* < 0.05). **G** Representative immunoblots and quantification of the effect of 24-hr treatment with 10 µM MG132 on Ca_V_2.1 regulation by Pin1 in HEK293T cells. Normalized Ca_V_2.1 protein level (*n* = 5): DMSO + vector, 1.01 ± 0.13; DMSO + Pin1, 0.71 ± 0.07; MG132 + vector, 13.70 ± 3.91; MG132 + Pin1, 18.57 ± 4.01. Asterisk denotes significant difference (*, *P* < 0.05). The difference between the Ca_V_2.1 protein levels for “MG132 + vector” and “MG132 + Pin1” is statistically insignificant (*P* > 0.05)

In line with its effect on total protein level, Pin1, but not the R69L mutant, suppressed surface Ca_V_2.1 protein abundance (Fig. 3A), as well as reducing Ca_V_2.1 current amplitude (Fig. 3B). Conversely, ATRA treatment promoted surface Ca_V_2.1 protein level (Fig. 3C). Coexpression with either Pin1 or the Pin1-R96L mutant did not detectably affect human Ca_V_2.1 mRNA level in HEK293T cells (Fig. 4A). Likewise, shRNA knockdown of endogenous Pin1 expression failed to discernibly change rat Ca_V_2.1 mRNA level in cultured cortical neurons (Fig. 4A). These observations imply that Pin1 may affect Ca_V_2.1 proteostasis by enhancing protein degradation. Consistent with this idea, cycloheximide chase analyses revealed that coexpression with Pin1, but not the R96L mutant, dramatically reduced the protein half-life of Ca_V_2.1 from about 8.1 h to about 2.6 h (Fig. 4B; Suppl. Fig. S3A-C). In contrast, shRNA knockdown of endogenous Pin1 expression in HEK293T cells considerably increased Ca_V_2.1 protein half-life (Fig. 4C). Furthermore, Pin1, but not the R96L mutant, significantly enhanced Ca_V_2.1 ubiquitination (Fig. 4D). Conversely, shRNA knockdown of endogenous Pin1 expression in HEK293T cells markedly attenuated Ca_V_2.1 ubiquitination (Suppl. Fig. S3D).

We have previously demonstrated that, during ER-associated degradation, Ca_V_2.1 was attached to polyubiquitin chains formed by covalent linkages of lysines [24], also known as polyubiquitination [1, 46]. The presence of a smear of high-molecular-weight Ca_V_2.1 ubiquitination pattern in Fig. 4D suggests that Pin1 may augment Ca_V_2.1 polyubiquitination. To test this hypothesis, we employed a lysine-less ubiquitin mutant, Ub-K0, which renders polyubiquitination practically impossible and thus significantly increases the abundance of Ca_V_2.1 and other proteins [8, 15, 24, 25, 32, 81]. Figure 4E illustrates that, in the presence of Pin1, coexpression with Ub-K0 led to prominent up-regulation of Ca_V_2.1 protein level. In contrast, in the presence of Ub-K0, coexpression with Pin1 failed to noticeably alter Ca_V_2.1 protein expression. Similarly, Ub-K0 prevented the Ca_V_2.1 protein enhancement effect of ATRA, despite the chemical’s significant reduction of endogenous Pin1 level (Fig. 4F). Moreover, treatment with the proteasome inhibitor MG132 virtually obliterated the Ca_V_2.1 protein suppression effect of Pin1 (Fig. 4G). The foregoing data indicate that Pin1 effectively promotes polyubiquitination and proteasomal degradation of Ca_V_2.1. Consistent with this inference, the auxiliary α_2_δ and β subunits, which protect nascent Ca_V_ α1 subunits from ER-associated polyubiquitination and degradation [3, 11, 24, 66, 82], regulated Ca_V_2.1 proteostasis in an opposite manner from Pin1; in addition, Pin1 and auxiliary subunits appeared to operate independently during ER quality control of Ca_V_2.1 (Suppl. Fig. S4).

### Pin1 interacts with specific domains in CaV2.1 subunit

**Fig. 5:**
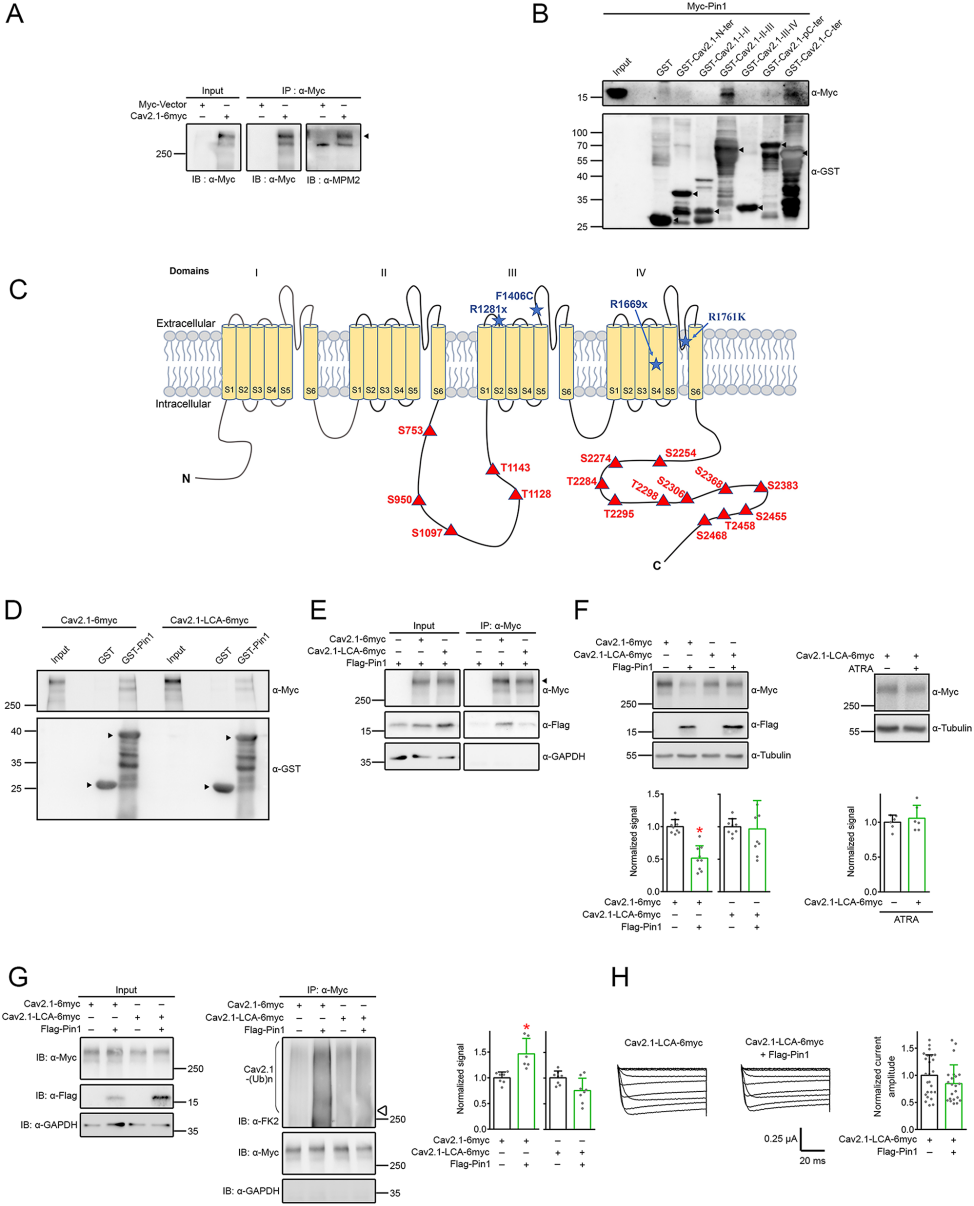
Identification of Pin-interacting domains in Ca_V_2.1. **A** Representative immunoblots showing the recognition of phospho-serine/threonine-proline (pSer/Thr-Pro) motifs in human Ca_V_2.1 long-isoform by the anti-Mitotic protein antibody α-MPM2. Lysates from transfected HEK293T cells were immunoprecipitated with α-Myc, followed by immunoblotting with α-Myc or α-MPM2. Arrowhead denotes the location of phosphorylated Ca_V_2.1 protein band at ~ 280 kDa. **B** Representative immunoblots demonstrating selective association of Myc-Pin1 with GST fusion proteins comprising human Ca_V_2.1 II-III loop (*Ca*_*V*_*2.1-II-III*) and distal carboxy-terminal fragment (*Ca*_*V*_*2.1-C-ter*), but not amino-terminal (*Ca*_*V*_*2.1-N-ter*), I-II loop (*Ca*_*V*_*2.1-I-II*), III-IV loop (*Ca*_*V*_*2.1-III-IV*), or proximal carboxy-terminal (*Ca*_*V*_*2.1-pC-ter*) regions. Lysates from HEK293T cells overexpressing Myc-Pin1 were subject to GST pull-down assay, followed by immunoblotting with α-Myc. Arrowheads denote the location of GST or GST-Ca_V_2.1 fusion protein bands. **C** Schematic membrane topology of human long carboxy-terminal Ca_V_2.1 subunit. Potential serine/threonine residues conforming to Pin1-recognizing pSer/Thr-Pro motifs in the II-III loop and the distal carboxy-terminal region are marked in *red*. Four known EA2-causing Ca_V_2.1 mutations, both nonsense (*R1281x*,* R1669x*) and missense (*F1406C*,* E1761K*), are labeled in *blue*. **D** Representative immunoblots illustrating the deficient interaction between the GST fusion protein comprising human Pin1 (*GST-Pin1*) and the human Ca_V_2.1-LCA construct, in which all the 16 serine/threonine residues at the potential pSer/Thr-Pro motifs in the II-III loop and the distal carboxy-terminal region of Ca_V_2.1 long-isoform were replaced with alanine. Lysates from HEK293T cells overexpressing Ca_V_2.1-6myc or Ca_V_2.1-LCA-6myc were subject to GST pull-down assay with GST or GST-Pin1, followed by immunoblotting with α-Myc. Arrowheads denote the location of GST or GST-Pin1 fusion protein bands. **E** Representative immunoblots depicting the deficient coimmunoprecipitation of Pin1 with Ca_V_2.1-LCA. Lysates from HEK293T cells coexpressing Flag-Pin1 with Myc vector (−), Ca_V_2.1-6myc, or Ca_V_2.1-LCA-6myc were immunoprecipitated with α-Myc, followed by immunoblotting with α-Myc and α-Flag. Arrowhead denotes the primary Ca_V_2.1 protein band. **F** Lack of effect of Pin1 and ATRA on Ca_V_2.1-LCA protein expression in HEK293T cells. (*Left panels*) Representative immunoblots and quantification of the effect of Pin1 coexpression (*n* = 9). Normalized Ca_V_2.1 protein level: vector, 1.00 ± 0.11; Pin1, 0.51 ± 0.19. Normalized Ca_V_2.1-LCA protein level: vector, 1.00 ± 0.12; Pin1, 0.97 ± 0.44. Asterisks denote significant difference from the cognate vector control (*, *P* < 0.05). (*Right panels*) Representative immunoblots and quantification of the effect of treatment with 50 µM ATRA. Normalized Ca_V_2.1-LCA protein level (*n* = 6): DMSO, 1.00 ± 0.10; ATRA, 1.06 ± 0.19. **G** Lack of effect of Pin1 on polyubiquitination of Ca_V_2.1-LCA. (*Left panels*) Representative immunoblots displaying Ca_V_2.1 and Ca_V_2.1-LCA polyubiquitination by endogenous ubiquitin in HEK293T cells. Open triangle refers to the expected location of the protein band corresponding to non-ubiquitinated Ca_V_2.1-LCA. (*Right panels*) Quantification of relative ubiquitination signals, which were normalized with respect to the corresponding Flag vector control. Normalized Ca_V_2.1 ubiquitination signal (*n* = 6–7): vector, 1.00 ± 0.11; Pin1, 1.47 ± 0.30. Normalized Ca_V_2.1-LCA ubiquitination signal (*n* = 8): 1.01 ± 0.13, Pin1, 0.76 ± 0.24. Asterisk denotes significant difference from the cognate vector control (*, *P* < 0.05). The difference between the Ca_V_2.1-LCA ubiquitination signals for “vector” and “Pin1” is statistically insignificant (*P* > 0.05). **H** Lack of effect of Pin1 on functional expression of Ca_V_2.1-LCA in *Xenopus* oocytes. (*Left panels*) Representative Ba^2+^ current traces through the Ca_V_2.1-LCA channel in the absence or presence of Pin1. Oocytes coinjected with Ca_V_2.1-LCA cRNA and water were used as the control. (*Right panels*) Quantification of relative peak Ba^2+^ current amplitude at + 20 mV (*n* = 22–25): control, 1.00 ± 0.38; Pin1, 0.85 ± 0.35. This difference is statistically insignificant (*P* > 0.05)

Pin1, a proline-directed foldase, catalyzes the *cis/trans* isomerization of peptidyl-prolyl bonds in a phosphorylation-dependent manner that requires phosphorylation of specific serine/threonine-proline (pSer/Thr-Pro) motifs in substrates [52, 53, 89]. We therefore applied a specific anti-phospho-Ser/Thr-Pro antibody, the anti-mototic protein antibody (α-MPM2), to determine whether Ca_V_2.1 may contain the Pin1-recognizing pSer/Thr-Pro motif. As shown in Fig. 5A, the MPM2 antibody recognized the 280-kDa Ca_V_2.1 protein band, indicating that human Ca_V_2.1 is indeed subject to effective phosphorylation at the specific serine/threonine-proline motif. In addition, GST pull-down analyses with fusion proteins comprising distinct intracellular domains of Ca_V_2.1 suggested that Pin1 primarily binds to the intracellular loop segment connecting transmembrane domains II and III (II-III loop; amino acids: 715–1245), as well as the cytoplasmic distal carboxy-terminal region (amino acids: 2204–2510), of the long carboxy-terminal isoform of human Ca_V_2.1 subunit (Fig. 5B).

Figure 5C depicts the schematic location of the potential Pin1-recognizing pSer/Thr-Pro motifs located in the II-III loop (five sites) and the distal carboxy-terminal region (11 sites) of the long isoform of human Ca_V_2.1. To address the significance of these pSer/Thr-Pro motifs in Pin1-Ca_V_2.1 interaction, we constructed a protein fragment corresponding to the distal carboxy-terminal region of Ca_V_2.1 (the dCT fragment). Similar to the results obtained from full-length Ca_V_2.1, Pin1 interacted with and reduced protein expression of the dCT fragment (Suppl. Fig. S5A-C), whereas suppression of endogenous Pin1 activity promoted dCT level (Suppl. Fig. S5E-F). We then generated a series of different mutations in the dCT fragment, wherein the serine/threonine residues at the 11 potential Pin1-recognizing pSer/Thr-Pro sites were systemically mutated into alanine (Suppl. Fig. S5A-D). Importantly, replacing all 11 serine/threonine residues with alanine in the dCT fragment (dCT-11 A) virtually abolished the binding and regulatory effects of Pin1 (Suppl. Fig. S5). Further mutation analyses identified four essential Pin1-interacting serine/threonine residues in the dCT fragment (dCT-4 A) (Suppl. Fig. S5). Nevertheless, introduction of either the four essential or all 11 distal carboxy-terminal alanine substitutions into full-length Ca_V_2.1 (Ca_V_2.1-C4A, Ca_V_2.1-C11A) per se did not noticeably attenuate the regulatory effect of Pin1 (Suppl. Fig. S6), implying a crucial role of the five Pin1-recognizing pSer/Thr-Pro motifs in the II-III loop.

We therefore generated another Ca_V_2.1 construct, Ca_V_2.1-LCA, in which all the serine/threonine residues at the pSer/Thr-Pro motifs in the II-III loop and the distal carboxy-terminal region were replaced with alanine. The GST-Pin1 binding affinity as well as the Pin1 coimmunoprecipitation efficiency of Ca_V_2.1-LCA was significantly lower compared to its WT counterpart (Fig. 5D-E). Importantly, Pin1 failed to detectably reduce protein level or promote polyubiquitination of Ca_V_2.1-LCA, and suppression of endogenous Pin1 level with ATRA did not discernibly increase Ca_V_2.1-LCA protein expression (Fig. 5F-G). Figure 5H further demonstrates that Ca_V_2.1-LCA produced functional Ca^2+^ channel, and that coexpression with Pin1 did not measurably decrease Ca_V_2.1-LCA current level. Collectively, our data support the idea that the II-III loop and the distal carboxy-terminal region serve as the primary Pin1-interacting domains of human Ca_V_2.1.

### Pin1-mediated isomerization is necessary for CaV2.1 degradation by RNF138

**Fig. 6:**
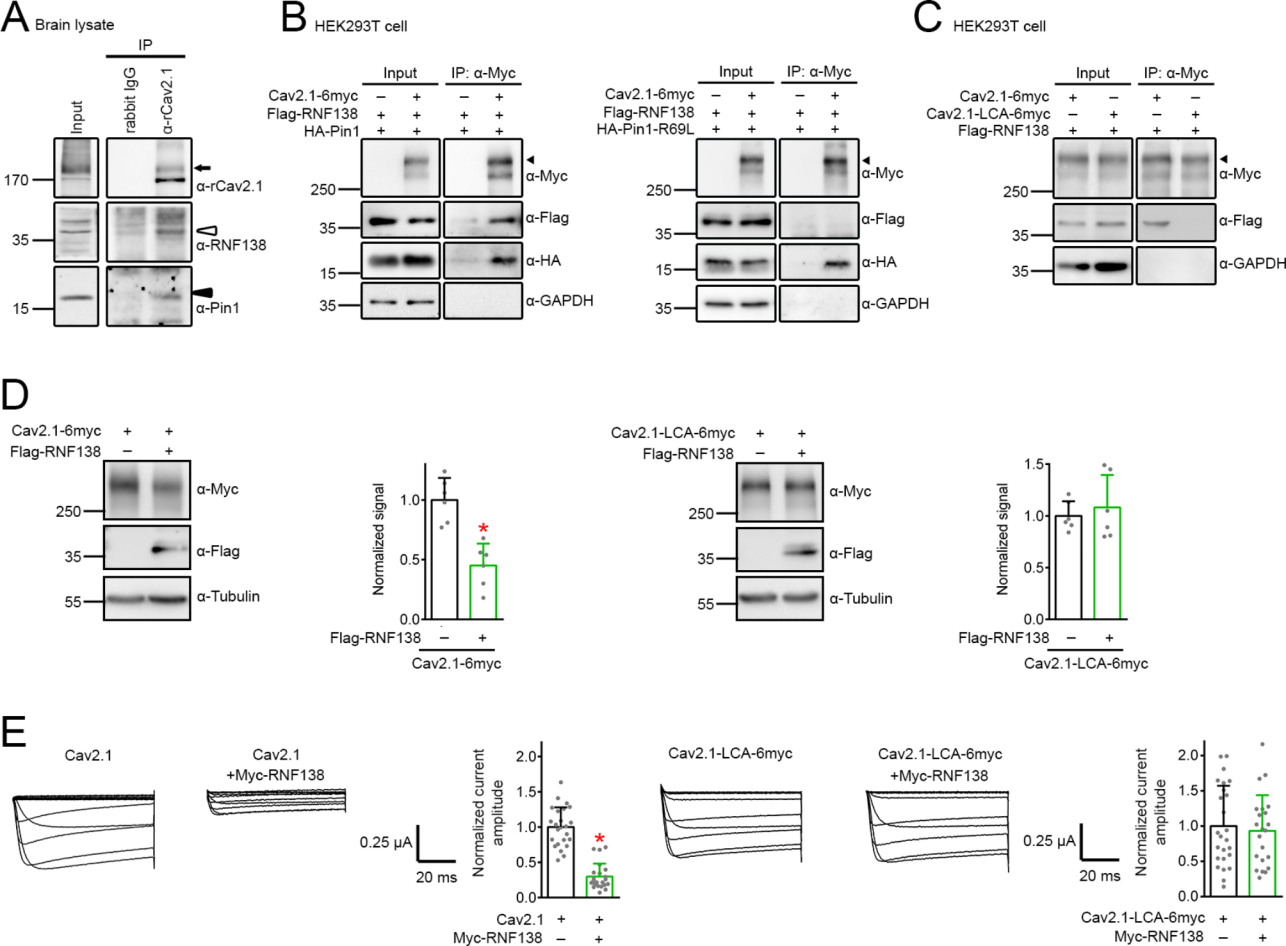
Requirement of Pin1-mediated isomerization for Ca_V_2.1 degradation by RNF138. **A** Representative immunoblots showing the coimmunoprecipitation of endogenous Ca_V_2.1 (*arrow*), RNF138 (*open triangle*), and Pin1 (*arrowhead*) in the rat brain. Rat forebrain lysates were immunoprecipitated with α-rCa_V_2.1 or the rabbit IgG. **B** Representative immunoblots demonstrating the coimmunoprecipitation of RNF138 with Ca_V_2.1 and Pin1 (*left*), but not Ca_V_2.1 and the isomerase-inactive mutant Pin1-R69L (*right*). Lysates from HEK293T cells coexpressing Flag-RNF138 with Myc vector (−), Ca_V_2.1-6myc, HA-Pin1, or HA-Pin1-R69L were immunoprecipitated with α-Myc, followed by immunoblotting with α-Myc, α-Flag, and α-HA. Arrowheads denote the primary Ca_V_2.1 protein band. **C** Representative immunoblots depicting the deficient coimmunoprecipitation of RNF138 with Ca_V_2.1-LCA. Lysates from HEK293T cells coexpressing Flag-RNF138 with Myc vector (−), Ca_V_2.1-6myc, or Ca_V_2.1-LCA-6myc were immunoprecipitated with α-Myc, followed by immunoblotting with α-Myc and α-Flag. Arrowhead denotes the primary Ca_V_2.1 protein band. **D** Lack of effect of RNF138 on Ca_V_2.1-LCA protein expression in HEK293T cells. Representative immunoblots and quantification of the effect of RNF138 coexpression on Ca_V_2.1 (*left panels*) or Ca_V_2.1-LCA (*right panels*). Normalized Ca_V_2.1 protein level (*n* = 6): vector, 1.00 ± 0.18; RNF138, 0.45 ± 0.18. Normalized Ca_V_2.1-LCA protein level (*n* = 6): vector, 1.00 ± 0.14; RNF138, 1.08 ± 0.31. Asterisk denotes significant difference from the cognate vector control (*, *P* < 0.05). **E** Lack of effect of RNF138 on functional expression of Ca_V_2.1-LCA in *Xenopus* oocytes. (*Left panels*) Representative Ba^2+^ current traces and quantification of the effect of RNF138 coexpression on Ca_V_2.1 (*left panels*) or Ca_V_2.1-LCA (*right panels*). Oocytes coinjected with Ca_V_2.1/Ca_V_2.1-LCA cRNA and water were used as the control. Normalized Ca_V_2.1 current amplitude at + 20 mV (*n* = 22–24): control, 1.00 ± 0.28; RNF138, 0.30 ± 0.19. Normalized Ca_V_2.1-LCA current amplitude at + 20 mV (*n* = 22–25): control, 1.00 ± 0.57; RNF138, 0.93 ± 0.50. Asterisk denotes significant difference from the cognate control (*, *P* < 0.05)

During membrane protein biogenesis in the ER, PPIases, along with molecular chaperones and other foldases, facilitate proper folding of nascent protein chains into native conformations prior to their exit from the ER; furthermore, E3 ubiquitin ligases assist the disposal of misfolded proteins from the ER to the proteasome [9, 16, 22]. Based on the foregoing findings, we deduced that, as a PPIase, Pin1 might catalyze peptidyl-prolyl *cis/trans* isomerization of Ca_V_2.1, and thereby enhance the interaction of misfolded Ca_V_2.1 with E3 ubiquitin ligases such as RNF138, which we have previously shown to play a critical role in ER-associated degradation of Ca_V_2.1 WT and EA2-causing mutants [24]. In agreement with this hypothesis, we observed that, in rat brain lysates, endogenous Ca_V_2.1, Pin1, and RNF138 coexisted in the same protein complex (Fig. 6A). Likewise, in the HEK293T overexpression system, RNF138 was effectively recruited to the Ca_V_2.1-Pin1 complex (Fig. 6B). Upon replacing Pin1 with the isomerase-inactive mutant Pin1-R69L, however, RNF138 failed to coexist with the Ca_V_2.1-Pin1-R69L complex (Fig. 6B). In addition, unlike Ca_V_2.1, the Pin1-insensitive Ca_V_2.1-LCA was not detectably coimmunoprecipitated with RNF138 (Fig. 6C). Most importantly, coexpression with RNF138 did not appear to affect either protein level or functional expression of Ca_V_2.1-LCA (Fig. 6D-E). Similarly, the Pin1-insensitive Ca_V_2.1 dCT-4 A and dCT-11 A fragments failed to be detectably regulated by RNF138, either (Suppl. Fig. S7). Overall, these observations are consistent with the notion that, during ER quality control, Pin1 serves as an upstream regulator of Ca_V_2.1-RNF138 interaction, and that Pin1-catalyzed *cis/trans* isomerization dictates the extent of Ca_V_2.1 polyubiquitination and degradation by RNF138.

### Pin1 contributes to EA2-related anomalous CaV2.1 proteostasis

**Fig. 7:**
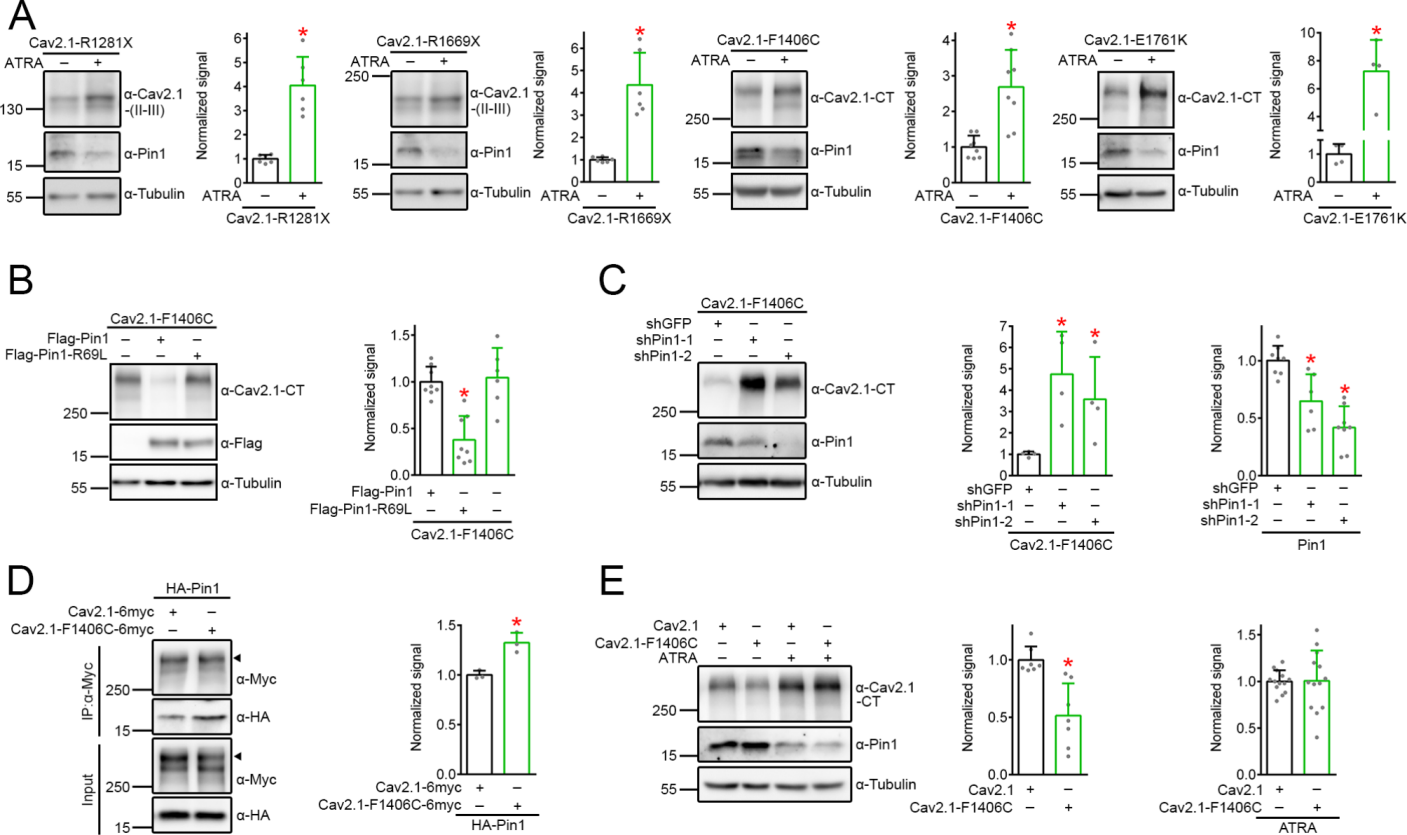
Proteostatic regulation of EA2-causing Ca_V_2.1 nonsense and missense mutants by Pin1 in HEK293T cells. **A** Representative immunoblots and quantification of the effect of ATRA on EA2-causing Ca_V_2.1 nonsense (*R1281x*,* R1669x*) and missense (*F1406C*,* E1761K*) mutants. Lysates from transfected cells were subject to treatment with DMSO or 50 µM ATRA for 24 h. Normalized Ca_V_2.1-R1281x protein level (*n* = 6): DMSO, 1.00 ± 0.17; ATRA, 4.05 ± 1.19. Normalized Ca_V_2.1-R1669x protein level (*n* = 6): DMSO, 1.00 ± 0.11, ATRA, 4.35 ± 1.45. Normalized Ca_V_2.1-F1406C protein level (*n* = 6): DMSO, 1.00 ± 0.32; ATRA, 2.69 ± 1.05. Normalized Ca_V_2.1-E1761K protein level (*n* = 5): DMSO, 1.00 ± 0.36; ATRA, 7.25 ± 2.24. Asterisks denote significant difference from the cognate DMSO control (*, *P* < 0.05). **B** Representative immunoblots and quantification of the effect of Pin1 and Pin1-R69L on Ca_V_2.1-F1406C. Normalized Ca_V_2.1-F1406C protein level (*n* = 7–8): vector, 1.00 ± 0.16; Pin1, 0.38 ± 0.26; Pin1-R69L, 1.05 ± 0.32. Asterisk denotes significant difference from the vector control (*, *P* < 0.05). **C** Representative immunoblots and quantification of the effect of shRNA knockdown of endogenous Pin1 on Ca_V_2.1-F1406C. Normalized Ca_V_2.1-F1406C protein level (*n* = 4): shGFP, 1.00 ± 0.12; shPin1-1, 4.74 ± 2.00; shPin1-2, 3.58 ± 1.98. Normalized endogenous Pin1 protein level (*n* = 6–8): shGFP, 1.00 ± 0.13; shPin1-1, 0.65 ± 0.23; shPin1-2, 0.42 ± 0.19. Asterisks denote significant difference from the shGFP control (*, *P* < 0.05). **D** Representative immunoblots and quantification of the Pin1 coimmunoprecipitation efficiency of Ca_V_2.1 and Ca_V_2.1-F1406C. Lysates from cells coexpressing HA-Pin1 with Ca_V_2.1 or Ca_V_2.1-F1406C were subject to treatment with 10 µM MG132 for 24 h, followed by immunoprecipitation with α-Myc and immunoblotting analyses with α-Myc and α-HA. Arrowheads denote the primary Ca_V_2.1 protein band. Normalized coimmunoprecipitated Pin1 protein level (*n* = 3): Ca_V_2.1, 1.00 ± 0.04; Cav2.1-F1406C, 1.32 ± 0.10. Asterisk denotes significant difference from Ca_V_2.1 (*, *P* < 0.05). **E** Representative immunoblots and quantification of the effect of ATRA on relative protein expression of Ca_V_2.1 and Ca_V_2.1-F1406C. Normalized protein level in response to DMSO (*n* = 7): Ca_V_2.1, 1.00 ± 0.12; F1406C, 0.51 ± 0.28. Normalized protein level in response to ATRA (*n* = 13–14): Ca_V_2.1, 1.00 ± 0.12; Ca_V_2.1-F1406C, 1.01 ± 0.32. Asterisk denotes significant difference from Ca_V_2.1 (*, *P* < 0.05)

Many of the EA2-causing nonsense and missense mutations are associated with defected proteostasis, manifesting as enhanced protein degradation and/or impaired membrane trafficking of human Ca_V_2.1 [24, 39, 59, 83]. To determine whether Pin1 may contribute to EA2-aasociated defective proteostasis, we began by testing the effect of Pin1 on protein expression of Ca_V_2.1 nonsense (R1281x and R1669x) and missense (F1406C, E1761K) mutants [17, 19, 35, 91] (see Fig. 5C for schematic locations of the mutants). Figure 7A illustrates that reduction of endogenous Pin1 level with ATRA substantially promoted protein expression of the four Ca_V_2.1 EA2 mutants, suggesting that these mutants are subject to Pin1 regulation. We then focused on the missense mutation F1406C, a domain-III pore-loop mutant that produced small but detectable ionic currents [35, 38], for further validation of the proteostatic significance of Pin1 in EA2. Similar to its WT counterpart, coexpression with Pin1, but not the R69L mutant, significantly reduced F1406C protein level (Fig. 7B); conversely, shRNA knockdown of endogenous Pin1 expression notably increased F1406C protein abundance (Fig. 7C). Given that the F1406C mutant is subject to enhanced proteasomal degradation [24], the proteasomal inhibitor MG132 effectively rescued its defective protein level (Suppl. Fig. S8). Interestingly, in the presence of MG132, F1406C displayed higher Pin1 coimmunoprecipitation efficiency than did Ca_V_2.1 WT (Fig. 7D), suggesting that the EA2 mutant may be more susceptible to Pin1 regulation. Furthermore, upon ATRA treatment, the protein expression of F1406C was virtually identical with that of WT (Fig. 7E). Collectively, our data are consistent with the idea that Pin1 also plays an essential role in the defective proteostasis of EA2-causing Ca_V_2.1 nonsense and missense mutants.

### Pin1 is critical for dominant-negative effects of EA2 missense mutants on CaV2.1 WT

**Fig. 8:**
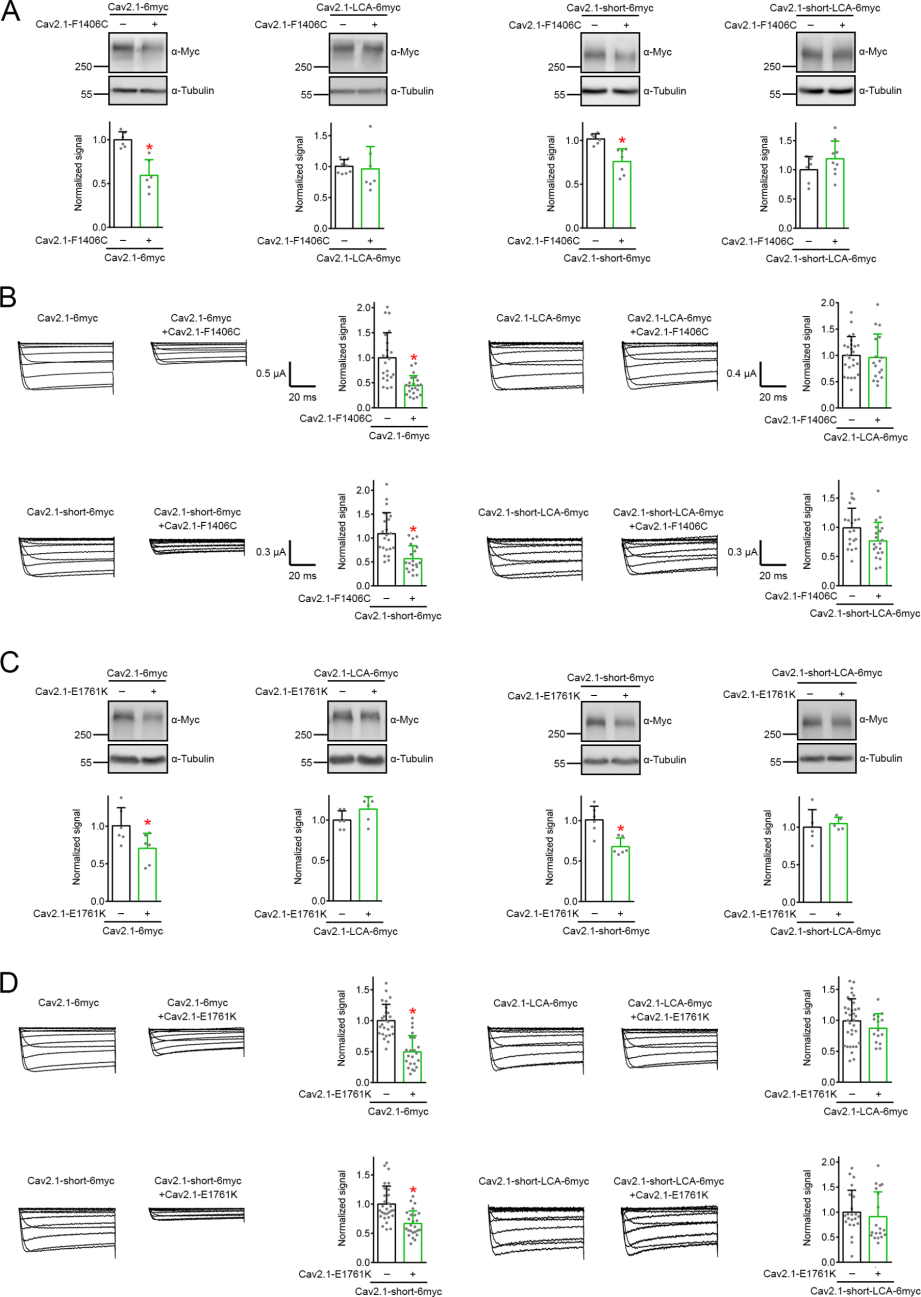
Contribution of Pin1 to the dominant-negative effect of EA2-causing Ca_V_2.1 missense mutants. **A**-**B** Lack of effect of Ca_V_2.1-F1406C coexpression on the Pin1-insenstive Ca_V_2.1-LCA and Ca_V_2.1-short-LCA protein level in HEK293T cells (**A**), and functional expression in *Xenopus* oocytes (**B**). **A** (*Left panels*) Normalized Ca_V_2.1 WT protein level (*n* = 6): vector, 1.00 ± 0.09; Ca_V_2.1-F1406C, 0.59 ± 0.18. Normalized Ca_V_2.1-LCA protein level (*n* = 7–9): vector, 1.00 ± 0.11; Ca_V_2.1-F1406C, 0.96 ± 0.36. (*Right panels*) Normalized Ca_V_2.1-short WT protein level (*n* = 6–7): vector, 1.02 ± 0.06; Ca_V_2.1-F1406C, 0.76 ± 0.15. Normalized Ca_V_2.1-short-LCA protein level (*n* = 6–9): vector, 1.00 ± 0.23; Ca_V_2.1-F1406C, 1.19 ± 0.30. **B** (*Top panels*) Normalized Ca_V_2.1 WT current amplitude at + 20 mV (*n* = 22): control, 1.00 ± 0.50; Ca_V_2.1-F1406C, 0.45 ± 0.19. Normalized Ca_V_2.1-LCA current amplitude at + 20 mV (*n* = 17–25): control, 1.00 ± 0.36; Ca_V_2.1-F1406C, 0.96 ± 0.45. (*Bottom panels*) Normalized Ca_V_2.1-short WT current amplitude at + 20 mV (*n* = 19–22): control, 1.09 ± 0.44; Ca_V_2.1-F1406C, 0.57 ± 0.27. Normalized Ca_V_2.1-short-LCA current amplitude at + 20 mV (*n* = 19–20): control, 1.01 ± 0.34; Ca_V_2.1-F1406C, 0.77 ± 0.32. **C**-**D** Lack of effect of Ca_V_2.1-E1761K coexpression on protein level (**C**) and functional expression (**D**) of Ca_V_2.1-LCA and Ca_V_2.1-short-LCA. **C** (*Left panels*) Normalized Ca_V_2.1 WT protein level (*n* = 6): vector, 1.01 ± 0.24; Ca_V_2.1-E1761K, 0.71 ± 0.20. Normalized Ca_V_2.1-LCA protein level (*n* = 6): vector, 1.00 ± 0.11; Ca_V_2.1-E1761K, 1.14 ± 0.15. (*Right panels*) Normalized Ca_V_2.1-short WT protein level (*n* = 6): vector, 1.01 ± 0.17; Ca_V_2.1-E1761K, 0.68 ± 0.11. Normalized Ca_V_2.1-short-LCA protein level (*n* = 5): vector, 1.00 ± 0.23; Ca_V_2.1-E1761K, 1.05 ± 0.08. **D** (*Top panels*) Normalized Ca_V_2.1 WT current amplitude at + 20 mV (*n* = 22–26): control, 1.00 ± 0.27; Ca_V_2.1-E1761K, 0.50 ± 0.26. Normalized Ca_V_2.1-LCA current amplitude at + 20 mV (*n* = 15–34): control, 1.00 ± 0.36; Ca_V_2.1-E1761K, 0.87 ± 0.23. (*Bottom panels*) Normalized Ca_V_2.1-short WT current amplitude at + 20 mV (*n* = 25–29): control, 1.00 ± 0.31; Ca_V_2.1-E1761K, 0.67 ± 0.22. Normalized Ca_V_2.1-short-LCA current amplitude at + 20 mV (*n* = 19–22): control, 1.00 ± 0.44; Ca_V_2.1-E1761K, 0.91 ± 0.49. Asterisks denote significant difference from the cognate control (*, *P* < 0.05)

Many EA2-causing nonsense and missense mutants, including the aforementioned R1281x, F1406C, R1669x, and E1761K mutants, suppressed functional expression of their WT counterpart via disruption of Ca_V_2.1 proteostasis [18, 21, 24, 38, 39, 42, 59, 64, 65, 69]. At least for nonsense mutants, this dominant-negative effect may involve a suppressive interaction between amino-terminal sequences of EA2 mutants and Ca_V_2.1 WT, thereby disrupting protein folding of Ca_V_2.1 WT [18]. It is thus likely that EA2-related missense mutants may also physically interact with and prevent proper folding of their WT counterpart. A key question then is whether Pin1 takes part in the dominant-negative effect of EA2 missense mutants on Ca_V_2.1 proteostasis. To address this issue, we first compared the effect of F1406C coexpression on Ca_V_2.1 WT and the Pin1-insensitive Ca_V_2.1-LCA. Unlike its well-characterized dominant-negative effects on Ca_V_2.1 WT, F1406C failed to discernibly affect either protein level or current amplitude of Ca_V_2.1-LCA (Fig. 8A-B).

So far, we mainly focused on the long carboxy-terminal isoform of Ca_V_2.1, which accounts for about two-thirds of *CACNA1A* transcripts in the human cerebellum [77]. As mentioned above, both the long and the short carboxy-terminal isoforms of human Ca_V_2.1 were subject to Pin1 regulation (Fig. 2A-B). Importantly, EA2 nonsense and missense mutants exerted significant dominant-negative effect on proteostasis of Ca_V_2.1-short as well [59]. We thus generated a modified Ca_V_2.1-short construct, Ca_V_2.1-short-LCA, in which all the equivalent, serine/threonine residues at the Pin1-recognizing pSer/Thr-Pro motifs in the II-III loop and the distal carboxy-terminal region were replaced with alanine. Similar to its long isoform counterpart (Ca_V_2.1-LCA), Ca_V_2.1-short-LCA appeared to be insensitive to Pin1 regulation (Suppl. Fig. S9). Upon coexpression with F1406C, both the protein level and current amplitude of Ca_V_2.1-short-LCA did not seem to be visibly affected (Fig. 8A-B). Figure 8C and D further highlight that the EA2-causing missense mutant E1761K also failed to exert significant dominant-negative effect on Ca_V_2.1-LCA and Ca_V_2.1-short-LCA. Taken as a whole, these observations imply that Pin1 regulation is essential for the dominant-negative effect of EA2 missense mutants on Ca_V_2.1 WT.

### Pin1 is not required for dominant-negative effects of EA2 nonsense mutants

**Fig. 9:**
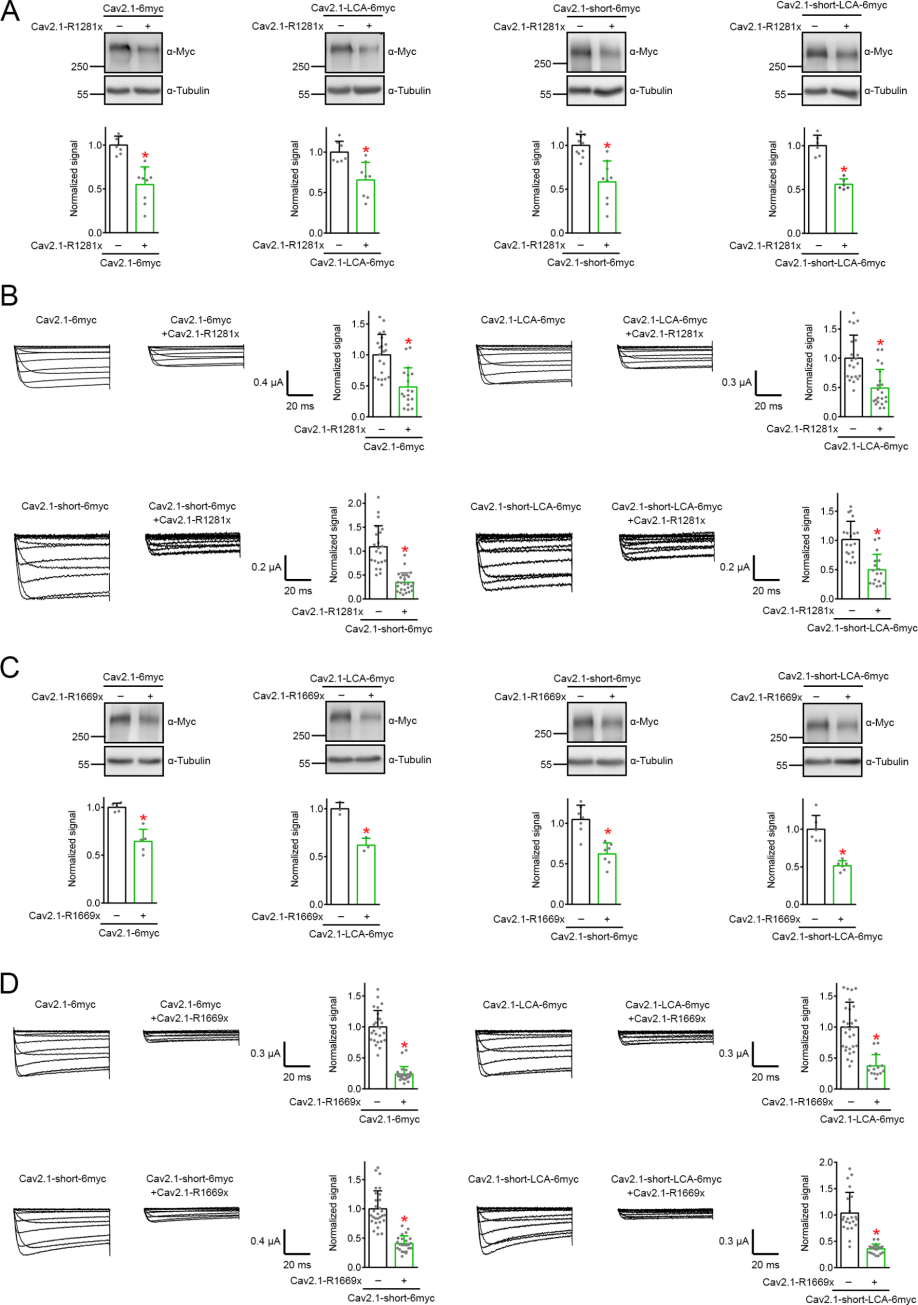
Proteostatic suppression of Pin1-insenstive Ca_V_2.1 constructs by EA2-causing nonsense mutants. **A**-**B** Dominant-negative effect of Ca_V_2.1-R1281x on Ca_V_2.1-LCA and Ca_V_2.1-short-LCA protein level in HEK293T cells (**A**), as well as functional expression in *Xenopus* oocytes (**B**). **A** (*Left panels*) Normalized Ca_V_2.1 WT protein level (*n* = 7–9): vector, 1.00 ± 0.10; Ca_V_2.1-R1281x, 0.55 ± 0.20. Normalized Ca_V_2.1-LCA protein level (*n* = 7–8): vector, 1.00 ± 0.13; Ca_V_2.1-R1281x, 0.66 ± 0.22. (*Right panels*) Normalized Ca_V_2.1-short WT protein level (*n* = 9–11): vector, 1.00 ± 0.12; Ca_V_2.1-R1281x, 0.58 ± 0.24. Normalized Ca_V_2.1-short-LCA protein level (*n* = 6): vector, 1.00 ± 0.12; Ca_V_2.1-R1281x, 0.56 ± 0.06. **B** (*Top panels*) Normalized Ca_V_2.1 WT current amplitude at + 20 mV (*n* = 18–21): control, 1.00 ± 0.33; Ca_V_2.1-R1281x, 0.48 ± 0.31. Normalized Ca_V_2.1-LCA current amplitude at + 20 mV (*n* = 21): control, 1.00 ± 0.39; Ca_V_2.1-R1281x, 0.49 ± 0.32. (*Bottom panels*) Normalized Ca_V_2.1-short WT current amplitude at + 20 mV (*n* = 22–25): control, 1.09 ± 0.44; Ca_V_2.1-R1281x, 0.35 ± 0.20. Normalized Ca_V_2.1-short-LCA current amplitude at + 20 mV (*n* = 17–19): control, 1.02 ± 0.31; Ca_V_2.1-R1281x, 0.50 ± 0.26. **C**-**D** Dominant-negative effect of Ca_V_2.1-R1669x on protein level (**C**) and functional expression (**D**) of Ca_V_2.1-LCA and Ca_V_2.1-short-LCA. **C** (*Left panels*) Normalized Ca_V_2.1 WT protein level (*n* = 5): vector, 1.00 ± 0.04; Ca_V_2.1-R1669x, 0.64 ± 0.13. Normalized Ca_V_2.1-LCA protein level (*n* = 3): vector, 1.00 ± 0.07; Ca_V_2.1-R1669x, 0.62 ± 0.07. (*Right panels*) Normalized Ca_V_2.1-short WT protein level (*n* = 7): vector, 1.05 ± 0.18; Ca_V_2.1-R1669x, 0.62 ± 0.14. Normalized Ca_V_2.1-short-LCA protein level (*n* = 6): vector, 1.00 ± 0.18; Ca_V_2.1-R1669x, 0.51 ± 0.07. **D** (*Top panels*) Normalized Ca_V_2.1 WT current amplitude at + 20 mV (*n* = 26): control, 1.00 ± 0.27; Ca_V_2.1-R1669x, 0.24 ± 0.12. Normalized Ca_V_2.1-LCA current amplitude at + 20 mV (*n* = 14–30): control, 1.00 ± 0.40; Ca_V_2.1-R1669x, 0.37 ± 0.18. (*Bottom panels*) Normalized Ca_V_2.1-short WT current amplitude at + 20 mV (*n* = 24–29): control, 1.00 ± 0.31; Ca_V_2.1-R1669x, 0.41 ± 0.13. Normalized Ca_V_2.1-short-LCA current amplitude at + 20 mV (*n* = 20–22): control, 1.04 ± 0.39; Ca_V_2.1-R1669x, 0.36 ± 0.09. Asterisks denote significant difference from the cognate control (*, *P* < 0.05). **E-H** Dominant-negative effect of Ca_V_2.1-R1669x on protein level and functional expression of Ca_V_2.1-LCA (**E**-**F**) and Ca_V_2.1-short-LCA (**G**-**H**). (**E**) Normalized Ca_V_2.1 WT protein level (*n* = 5): vector, 1.00 ± 0.04; Ca_V_2.1-R1669x, 0.64 ± 0.13. Normalized Ca_V_2.1-LCA protein level (*n* = 3): vector, 1.00 ± 0.07; Ca_V_2.1-R1669x, 0.62 ± 0.07. Asterisk denotes significant difference from the cognate vector control (*, *P* < 0.05). (**F**) Normalized Ca_V_2.1 WT current amplitude at + 20 mV (*n* = 26): control, 1.00 ± 0.27; Ca_V_2.1-R1669x, 0.24 ± 0.12. Normalized Ca_V_2.1-LCA current amplitude at + 20 mV (*n* = 14–30): control, 1.00 ± 0.40; Ca_V_2.1-R1669x, 0.37 ± 0.18. Asterisk denotes significant difference from the cognate control (*, *P* < 0.05). (**G**) Normalized Ca_V_2.1-short WT protein level (*n* = 7): vector, 1.05 ± 0.18; Ca_V_2.1-R1669x, 0.62 ± 0.14. Normalized Ca_V_2.1-short-LCA protein level (*n* = 6): vector, 1.00 ± 0.18; Ca_V_2.1-R1669x, 0.51 ± 0.07. Asterisk denotes significant difference from the cognate vector control (*, *P* < 0.05). (**H**) Normalized Ca_V_2.1-short WT current amplitude at + 20 mV (*n* = 24–29): control, 1.00 ± 0.31; Ca_V_2.1-R1669x, 0.41 ± 0.13. Normalized Ca_V_2.1-short-LCA current amplitude at + 20 mV (*n* = 20–22): control, 1.04 ± 0.39; Ca_V_2.1-R1669x, 0.36 ± 0.09. Asterisks denote significant difference from the cognate control (*, *P* < 0.05)

Misfolded EA2-related nonsense mutants may trigger dominant-negative effects on Ca_V_2.1 WT by unfolded protein response [64], or by ER-associated degradation [24, 59]. Hence, we also asked whether Pin1 contributes to defective proteostasis of Ca_V_2.1 WT induced by EA2 nonsense mutants such as R1281x and R1669x. Coexpression with R1281x resulted in comparable reduction in protein level for both Ca_V_2.1 and Ca_V_2.1-LCA (Fig. 9A). In addition, R1281x displayed similar functional suppression effect on Ca_V_2.1 and Ca_V_2.1-LCA (Fig. 9B). Figure 9C and D further demonstrate that the Pin1-insensitive Ca_V_2.1-LCA was susceptible to the dominant-negative effect of the nonsense mutant R1669x. Both R1281x and R1669x substantially decreased protein level and current amplitude of Ca_V_2.1-short-LCA as well (Fig. 9). Therefore, it is likely that Pin1 regulation is dispensable for the dominant-negative effect of EA2 nonsense mutants.

## Discussion

In the current study, we aimed to ascertain the molecular mechanisms underlying ER quality control of human Ca_V_2.1 channels. The PPIase Pin1 was identified as a novel Ca_V_2.1 binding partner that promoted polyubiquitination and proteasomal degradation of Ca_V_2.1, but not Ca_V_1.2 or Ca_V_2.2. By use of Pin1-insensitive Ca_V_2.1 constructs, we further demonstrated that, during ER quality control, Pin1 was necessary for Ca_V_2.1 polyubiquitination and degradation by the E3 ubiquitin ligase RNF138. Pin1 also contributed to enhanced protein degradation associated with EA2-causing missense and nonsense mutants. Intriguingly, Pin1 regulation was required for the dominant-negative effect of EA2 missense mutants, but not nonsense mutants, on Ca_V_2.1 WT protein expression.

The ubiquitously expressed, proline-directed foldase Pin1 plays a pivotal role in regulating phosphorylation-dependent, *cis/trans* isomerization of substrates, thereby modulating their affinity to respective E3 ubiquitin ligases and determining the extent of their degradation by the ubiquitin-proteasome system [40, 50, 52, 89]. For example, Pin1-interaction promotes proteasomal degradation of the promyelocytic leukemia protein by the Cullin3 E3 ubiquitin ligase complex [90]; in contrast, Pin1-catalyzed isomerization reduces ubiquitination of the transcription factor Nanog [60]. We have previously shown that RNF138 mediates the ubiquitin-proteasome pathway of both Ca_V_2.1 WT and EA2 mutants, as well as serving as an essential E3 ubiquitin ligase promoting EA2 mutant-induced aberrant degradation of Ca_V_2.1 WT [24]. In this report, we present the new evidence indicating that Pin1-catalyzed *cis/trans* isomerization facilitates Ca_V_2.1 polyubiquitination and degradation by RNF138. As revealed by our mutation analyses, Pin1 interacts with specific phosphorylated serine/threonine-proline (pSer/Thr-Pro) motifs in the intracellular II-III loop and the distal carboxy-terminal region of human Ca_V_2.1, highlighting a potential role of proline-directed phosphorylation in regulating Ca_V_2.1 proteostasis.

Based on our data, we infer that at least two different types of ER-associated degradation can account for the dominant-negative suppression of EA2 mutants on Ca_V_2.1 WT. On one hand, misfolded EA2 missense mutants may promote Pin1-catalyzed, RNF138-mediated degradation of Ca_V_2.1 WT. On the other hand, misfolded EA2 nonsense mutants probably elicit either unfolded protein response of Ca_V_2.1 WT [18, 64, 65], or ER-associated degradation of Ca_V_2.1 WT [24, 39, 59, 69] independent of Pin1 regulation. In other words, Ca_V_2.1 proteostasis at the ER comprises both Pin1/RNF138-dependent and -independent mechanisms.

**Fig. 10:**
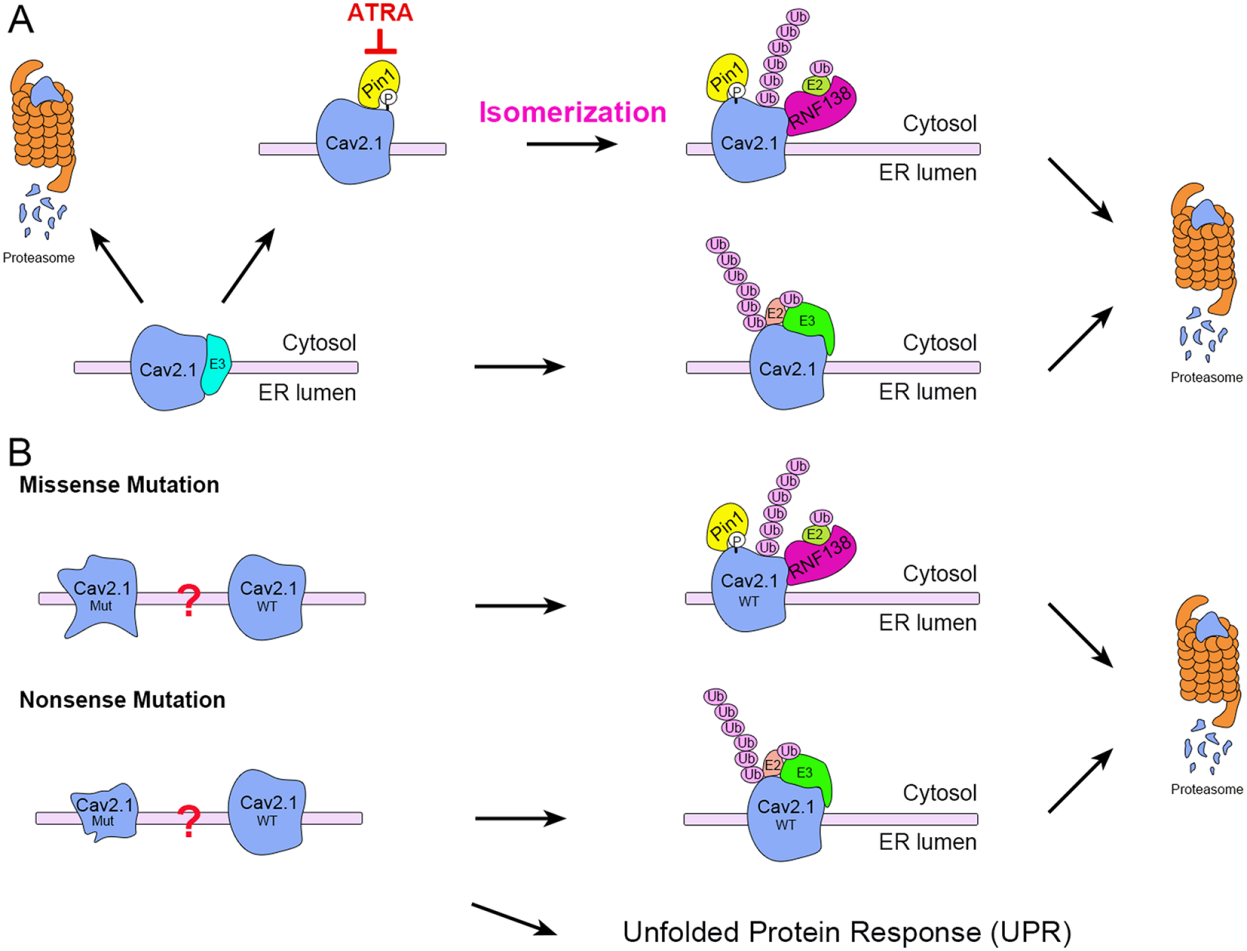
Schematic model of Pin1 regulation of human Ca_V_2.1 proteostasis at the ER. **A** ER quality control of Ca_V_2.1 is proposed to entail at least three sets of different protein folding checkpoints, each associated with a unique ER-associated degradation mechanism. Probably during translation or early protein folding stage, an upstream E3 ubiquitin ligase (*E3 in cyan*) might be recruited to promote proteasomal degradation of misfolded Ca_V_2.1. Subsequent post-translational folding at the ER might involve both Pin1-dependent and -independent pathways. The current study supports the idea that, following phosphorylation-dependent, Pin1-catalyzed *cis/trans* isomerization, misfolded Ca_V_2.1 is subject to polyubiquitination and proteasomal degradation mediated by the downstream E3 ligase RNF138. All-*trans* retinoic acid (*ATRA*), a therapeutic drug for leukemia, directly binds to and promotes degradation of Pin1, effectively preventing Ca_V_2.1 degradation by RNF138. On the other hand, in the Pin1-independent protein folding pathway, a distinct downstream E3 ligase (*E3 in green*) is speculated to target misfolded Ca_V_2.1 to the proteasome as well. **B** Dominant-negative effect of EA2-causing mutants is proposed to comprise both Pin1/RNF138-dependent and -independent ER quality control mechanisms. Through as yet unknown process (*red question mark*), misfolded EA2 missense mutants may interfere with protein folding of Ca_V_2.1 WT, and thereby facilitate Pin1-catalyzed isomerization and consequently enhance RNF138-mediated polyubiquitination and proteasomal degradation of Ca_V_2.1 WT. In contrast, perhaps via interaction with their amino-terminal regions (*red question mark*), misfolded EA2 nonsense mutants may disrupt either the upstream or the downstream Pin1/RNF138-independent protein folding steps of Ca_V_2.1 WT, leading to RNF138-independent proteasomal degradation, as well as unfolded protein response, of Ca_V_2.1 WT

Similar to the presence of multiple E3 ubiquitin ligases regulating proteostasis of the human chloride channel cystic fibrosis transmembrane conductance regulator (CFTR) [54, 68], E3 ubiquitin ligases other than RNF138 are likely to take part in the ER quality control mechanisms of human Ca_V_2.1 channels. We therefore propose that ER quality control of Ca_V_2.1 may start with an early, upstream Pin1/RNF138-independent protein folding checkpoint, followed by two distinct downstream protein folding-monitoring pathways involving Pin1/RNF138-dependent and -independent mechanisms (Fig. 10). In the downstream Pin1/RNF138-dependent ER quality control process, Pin1 and RNF138 play a critical role in determining Ca_V_2.1 protein turnover. In contrast, we speculate that both the upstream and the downstream Pin1/RNF138-independent ER quality control steps are mediated by a set of different E3 ubiquitin ligases. Through as yet unknown mechanisms, misfolded EA2 missense mutants (e.g., F1406C and E1781K) may substantially interfere with protein folding of their WT counterpart, resulting in increased Pin1-catalyzed *cis/trans* isomerization and consequently enhanced polyubiquitination and degradation by RNF138. On the contrary, perhaps by way of interaction with their amino-terminal regions [18], misfolded EA2 nonsense mutants (e.g., R1281x and R1669x) may predominantly disrupt either the upstream or the downstream Pin1/RNF138-independent protein folding steps of Ca_V_2.1 WT, leading to either unfolded protein response or RNF138-independent protein degradation. Future identification of additional Ca_V_2.1-targeting E3 ubiquitin ligases will help elucidate the molecular mechanisms underlying these Pin1/RNF138-independent ER quality control steps. Moreover, it will be imperative to establish how EA2-causing missense and nonsense mutants differentially affect protein folding of Ca_V_2.1 WT.

Both our biochemical and morphological analyses indicate that Ca_V_2.1 and Pin1 colocalize in the synaptic region of the rat brain, suggesting that, in addition to its regulatory role in ER quality control, Pin1 may additionally modulate Ca_V_2.1 proteostasis at neuronal plasma membrane. In the nervous system, Ca_V_2.1 channels critically control presynaptic neurotransmitter release; as well as postsynaptic dendritic Ca^2+^ transients and dendrosomatic excitability [13, 31, 67]. Likewise, Pin1 modulates multiple proteins essential for synaptic signaling [4, 5, 33, 75, 88, 92]. Nevertheless, it is still an open question whether Pin1 interaction may effectively affect Ca_V_2.1 channel function at either presynaptic terminals or postsynaptic compartments. Given that proteostasis of many synaptic signaling molecules are subject to extensive ubiquitin-mediated regulation [28, 47, 55–57, 74], further functional investigations are required to address the physiological significance of Pin1-catalyzed Ca_V_2.1 conformation change at synapses.

EA2 has long been associated with loss-of-function mutations in the human Ca_V_2.1-encoding *CACNA1A* gene [36, 37, 63, 67, 71]. Intriguingly, recent genetic studies further linked many loss-of-function *CACNA1A* variants to neurodevelopmental disorders such as developmental delay and developmental epileptic encephalopathy [23, 26, 29, 41, 43], highlighting the clinical significance of developing new treatment aiming at correcting defective Ca_V_2.1 proteostasis resulting from the dominant-negative effect of loss-of-function *CACNA1A* variants [41]. Herein we provided the novel evidence showing that ATRA, a potent Pin1 inhibitor well known for its therapeutic effect on leukemia and other cancer [44, 79, 86], effectively increases endogenous Ca_V_2.1 protein level in neurons, as well as notably reducing ER-associated degradation of EA2-causing Ca_V_2.1 mutants. These findings underscore the therapeutic potential of ATRA in alleviating neurological and neurodevelopmental disorders associated with defective Ca_V_2.1 proteostasis.

## Electronic supplementary material

Below is the link to the electronic supplementary material.

Supplementary Material 1: Supplementary figure S1 Representative confocal images of endogenous Ca_V_2.1 (*left panels*) and Pin1 (*right panels*) immunofluorescent signals in rat DIV10 cortical neurons. For each section, the boxed regions in the images shown in the upper rows are magnified for detailed inspection in the corresponding lower rows. **A** Localization of Ca_V_2.1 or Pin1 (*green*) in MAP2-positive (*red*) dendrites and somas, as highlighted by open triangles and further demonstrated in the merge images. **B** Localization of Ca_V_2.1 or Pin1 (*green*) in tau-positive (*red*) axons, as highlighted by arrows. **C** Colocalization of Ca_V_2.1 or Pin1 (*green*) with PSD95-puncta (*red*) along neurites, as denoted by arrowheads and further highlighted by yellow puncta in the merge images. Scale bars, 50 μm (*upper rows*) and 12.5 μm (*lower rows*)

Supplementary Material 2: Supplementary figure S2 Suppression of Ca_V_2.1 protein level by Pin1 overexpression. **A** Representative immunoblots verifying the specificity of the anti-Pin1 antibody, as well as comparing the relative apparent molecular weights of Flag-tagged rat Pin1 (*left panels*) and endogenous human Pin1. HEK293T cells were subject to shGFP or shPin1 infection (*right panels*) to validate the protein band corresponding to endogenous Pin1. **B** (*Top*) Representative immunoblot comparing Ca_V_2.1 protein levels in response to increasing coexpression ratios of Flag-Pin1 in HEK293T cells. The expression of tubulin is shown as the loading control. (*Bottom*) Quantification of relative Ca_V_2.1 protein levels with respect to Pin1 co-transfection ratios. Ca_V_2.1 signals were standardized as the ratio to the cognate tubulin signals, followed by normalization to the no Pin1 control, as well as single linear-regression analysis. Data were compiled from 3 independent experiments. **C** Representative immunoblot showing the effect of increasing the amount of Myc-Pin1 cDNA for transfection on endogenous Ca_V_2.1 (*arrowhead*) protein levels in HT-22 mouse hippocampal cells. Also shown are endogenous RNF138 and tubulin expression

Supplementary Material 3: Supplementary figure S3 Examination of Pin1 stability and the role of endogenous Pin1. **A-C** Protein turnover time course of Flag-tagged Pin1 and Pin1 R69L overexpressed in HEK293T cells. **A** Representative immunoblots. Transfected cells were subject cycloheximide (CHX) treatment for the indicated durations. **B** Quantification of Pin1 (*black*) and Pin1 R69L (*red*) protein turnover kinetics (*n* = 7). Protein density was normalized with respect to the corresponding value for no CHX treatment (0 h), followed by transformation into semilogarithmic plot and single linear-regression analyses. **C** Protein half-life values (hr): Pin1 (*black*), 16.40 ± 1.21; Pin1-R69L (*red*), 16.58 ± 1.41. **D** Representative immunoblots showing the effect of shRNA knockdown of endogenous Pin1 on human Ca_V_2.1 polyubiquitination in HEK293T cells. shGFP was used as the control. Lysates from cells overexpressing Ca_V_2.1-6myc were immunoprecipitated with α-Myc, followed by immunoblotting with the anti-ubiquitin antibody α-FK2. Ca_V_2.1 polyubiquitination [*Ca*_*V*_*2.1-(Ub)n*] by endogenous ubiquitin is visualized as high-molecular-weight protein smears. Normalized densitometric Ca_V_2.1 ubiquitination intensity is labeled on the immunoblot. Corresponding expression level of Ca_V_2.1, Pin1, and GAPDH in the lysates is shown in the *Input* lane

Supplementary Material 4: Supplementary figure S4 Independent regulation of human Ca_V_2.1 proteostasis by Pin1 and auxiliary subunits in HEK293T cells. **A**-**B** (*Top Panels*) Representative immunoblots showing the effect of the indicated auxiliary subunits on Ca_V_2.1 regulation by Pin1. Ca_V_2.1 was coexpressed with Pin1, α2δ, and β subunits in the molar ratios 1:3, 1:2, and 1:1, respectively. Coexpression with the Flag vector was used as the control. (*Bottom Panels*) Quantification of relative Ca_V_2.1 protein level (*n* = 5–6). Data were normalized with respect to the corresponding Flag vector control: (**A**) Pin1 + vector, 1.00 ± 0.10; Pin1 + β4a, 2.10 ± 0.67; Pin1 + β1b, 1.71 ± 0.36; Pin1 + β2a, 1.77 ± 0.43. (**B**) Pin1 + vector, 1.01 ± 0.10; Pin1 + α2δ, 1.94 ± 0.33; Pin1 + α2δ-β4a, 2.31 ± 0.90. **C** (*Top Panels*) Representative immunoblots showing the effect of Pin1 on Ca_V_2.1 regulation by the indicated auxiliary subunits. Coexpression with the Flag vector was used as the control. (*Bottom Panels*) Quantification of relative Ca_V_2.1 protein level (*n* = 5–6). Data were normalized with respect to the corresponding Flag vector control: (*left*) β4a + vector, 1.00 ± 0.10; β4a + Pin1, 0.38 ± 0.15. (center) α2δ + vector, 1.01 ± 0.13; α2δ + Pin1, 0.40 ± 0.12. (*right*) α2δ-β4a + vector, 1.00 ± 0.13; α2δ-β4a + Pin1, 0.62 ± 0.16. **D** Representative immunoblots and quantification of the effect of α2δ-β4a subunits on Pin1 regulation of Ca_V_2.1 protein stability. Ca_V_2.1 protein half-life values (hr) (*n* = 8): Pin1 + vector (*black*), 2.57 ± 1.00; Pin1 + α2δ-β4a (*red*), 9.54 ± 3.68. **E** Representative immunoblots and quantification of the effect of α2δ-β4a subunits on Pin1 regulation of Ca_V_2.1 polyubiquitination. Normalized Ca_V_2.1 ubiquitination signal (*n* = 5): Pin1 + vector, 1.01 ± 0.12; Pin1 + α2δ-β4a, 0.65 ± 0.18. Asterisks denote significant difference from the cognate vector control (*, *P* < 0.05)

Supplementary Material 5: Supplementary figure S5 Alanine mutation of potential Pin1-interacting serine/threonine residues in the distal carboxy-terminal (dCT) fragment of human Ca_V_2.1 long-isoform. *dCT*: no alanine mutation was carried out in the dCT fragment. *dCT-11 A*: all 11 potential Pin1-interacting serine/threonine residues (see Fig. 5C) were mutated into alanine. *dCT-4 A*: Four potential Pin1-interacting serine/threonine residues (corresponding to S2254, S2274, T2284, and S2383 in Fig. 5C) were mutated into alanine. *dCT-3/2/1A*: three/two/one potential Pin1-interacting serine/threonine residues were mutated into alanine. **A** Representative immunoblots showing the lack of interaction between GST-Pin1 and Myc-tagged dCT fragments harboring the indicated alanine mutations. Lysates from HEK293T cells overexpressing various dCT constructs were subject to GST pull-down assay with GST or GST-Pin1, followed by immunoblotting with α-Myc and α-GST. Arrowheads denote the location of GST or GST-Pin1 fusion protein bands. **B** Representative immunoblots demonstrating deficient coimmunoprecipitation of Pin1 with dCT fragments harboring the indicated alanine mutations. Lysates from HEK293T cells coexpressing Flag-Pin1 with various Myc-dCT constructs were subject to immunoprecipitation with α-Myc, followed by immunoblotting with α-Myc and α-Flag. **C-D** Lack of effect of Pin1 on protein expression of dCT-4 A and dCT-11 A in HEK293T cells. (*Top panels*) Representative immunoblots depicting the effect of coexpression with Flag-Pin1. Coexpression with Flag vector (−) was used as the control. (*Bottom panels*) Quantification of relative dCT protein level (*n* = 3–17). Asterisks denote significant difference from the cognate vector control (*, *P* < 0.05). **E-F** Lack of effect of Pin1-suppressing ATRA (**E**) and PiB (**F**) on protein expression of dCT-4 A and dCT-11 A in HEK293T cells. (*Left panels*) Representative immunoblots displaying the effect of treatment with 50 µM ATRA (**E**) and 10 µM PiB (**F**). Treatment with DMSO (−) was used as the control. (*Right panels*) Quantification of relative dCT protein level (*n* = 5–11). Asterisks denote significant difference from the cognate DMSO control (*, *P* < 0.05)

Supplementary Material 6: Supplementary figure S6 Pin1 regulation of human Ca_V_2.1 long-isoform harboring alanine substitution of either the 4 essential or all 11 distal carboxy-terminal Pin1-interacting serine/threonine residues (*Ca*_*V*_*2.1-C4A*,* Ca*_*V*_*2.1-C11A*). (*Top panels*) Representative immunoblots depicting the effect of coexpression with Flag-Pin1 on Ca_V_2.1 in HEK293T cells. Coexpression with Flag vector (−) was used as the control. (*Bottom panels*) Quantification of relative protein level. Normalized Ca_V_2.1 signal (*n* = 9): vector, 1.00 ± 0.12; Pin1, 0.51 ± 0.19. Normalized Ca_V_2.1-C4A signal (*n* = 7–9): vector, 1.01 ± 0.09; Pin1, 0.53 ± 0.19. Normalized Ca_V_2.1-C11A signal (*n* = 7–9): vector, 1.00 ± 0.10; Pin1, 0.62 ± 0.18. Asterisks denote significant difference from the cognate vector control (*, *P* < 0.05)

Supplementary Material 7: Supplementary figure S7 Lack of effect of RNF138 on Pin1-insenstive human Ca_V_2.1 dCT fragments harboring alanine substitution of either the 4 essential or all 11 Pin1-interacting serine/threonine residues (d*CT-4 A*,* dCT-11 A*). Representative immunoblots and quantification of the effect of RNF138 coexpression on the indicated dCT fragment constructs in HEK293T cells. Normalized dCT signal (*n* = 5–6): vector, 1.00 ± 0.09; RNF138, 0.65 ± 0.17. Normalized dCT-4 A signal (*n* = 5): vector, 1.00 ± 0.09; RNF138, 1.00 ± 0.24. Normalized dCT-11 A signal (*n* = 6): vector, 1.01 ± 0.06; RNF138, 0.92 ± 0.17. Asterisk denotes significant difference from the cognate vector control (*, *P* < 0.05)

Supplementary Material 8: Supplementary figure S8 Enhanced protreasomal degradation of Ca_V_2.1-F1406C. Representative immunoblots and quantification of the effect of the proteasomal inhibitor MG132 on relative protein expression of Ca_V_2.1 and Ca_V_2.1-F1406C. Lysates from transfected HEK293T cells were subject to treatment with DMSO or 10 µM MG132 for 24 h. Normalized protein level in response to DMSO (*n* = 6): Ca_V_2.1, 1.01 ± 0.13; Ca_V_2.1-F1406C, 0.43 ± 0.12. Normalized protein level in response to MG132 (*n* = 6): Ca_V_2.1, 1.01 ± 0.14; Ca_V_2.1-F1406C, 1.09 ± 0.18. Asterisk denotes significant difference from Ca_V_2.1 (*, *P* < 0.05)

Supplementary Material 9: Supplementary figure S9 Lack of effect of Pin1 on the human Ca_V_2.1 short-isoform construct harboring alanine substitution of all the potential Pin1-interacting serine/threonine residues in the II-III loop and the distal carboxy-terminal region (*Ca*_*V*_*2.1-short-LCA*). **A** Representative immunoblots and quantification of the effect of Pin1 on protein level of Ca_V_2.1-short and Ca_V_2.1-short-LCA in HEK293T cells. Data were normalized with respect to the corresponding vector control. Normalized Ca_V_2.1-short signal (*n* = 6): vector, 1.01 ± 0.12; Pin1, 0.54 ± 0.15. Normalized Ca_V_2.1-short-LCA signal (*n* = 6): vector, 1.00 ± 0.11; Pin1, 0.99 ± 0.09. Asterisk denotes significant difference from the cognate vector control (*, *P* < 0.05). **B** Representative Ba^2+^ current traces and quantification of the effect of Pin1 on functional expression of Ca_V_2.1-short and Ca_V_2.1-short-LCA in *Xenopus* oocytes. Data were normalized with respect to the corresponding water coinjection control. Normalized Ca_V_2.1-short current amplitude at + 20 mV (*n* = 15–16): control, 1.00 ± 0.34; Pin1, 0.59 ± 0.29. Normalized Ca_V_2.1-short-LCA current amplitude at + 20 mV (*n* = 13): control, 1.00 ± 0.31; Pin1, 0.89 ± 0.33. Asterisk denotes significant difference from the cognate control (*, *P* < 0.05)

## Data Availability

No datasets were generated or analysed during the current study.

## Abbreviations

ATRA: All-trans retinoic acid
Ca_V_2.1 channel: Voltage-gated P/Q-type Ca^2+^ channel
CHX: Cycloheximide
CFTR: Cystic fibrosis transmembrane conductance regulator
dCT: Distal carboxy-terminal
DIV: Days in vitro
D-PBS: Dulbecco’s phosphate buffered saline
DMEM: Dulbecco’s modified Eagle’s medium
DMSO: Dimethyl sulfoxide
DTT: Dithiothreitol
EA2: Episodic ataxia type 2
ER: Endoplasmic reticulum
GAPDH: Glyceraldehyde-3-phosphate dehydrogenase
GST: Glutathione S-transferase
HEK: Human embryonic kidney
IP: Immunoprecipitation
PBS: Phosphate buffered saline
Pin1: Peptidyl-prolyl cis/trans isomerase, NIMA-interacting 1
PMSF: Phenylmethylsulfonyl fluoride
PPIase: Peptidyl-prolyl cis/trans isomerase
PSD: Postsynaptic density
pSer/Thr-Pro: Phosphorylated serine/threonine-proline
RING: Really interesting new gene
shRNA: Short hairpin RNA
SPM: Synaptosomal
Ub: Ubiquitin
WT: Wild-type
