# Supplementary figures and images for: Pin1 promotes human CaV2.1 channel polyubiquitination by RNF138: pathophysiological implication for episodic ataxia type 2
Source: Cell Commun Signal. 2024 Nov 28;22:571. doi: 10.1186/s12964-024-01960-9 (PMC11603662; doi:10.1186/s12964-024-01960-9)

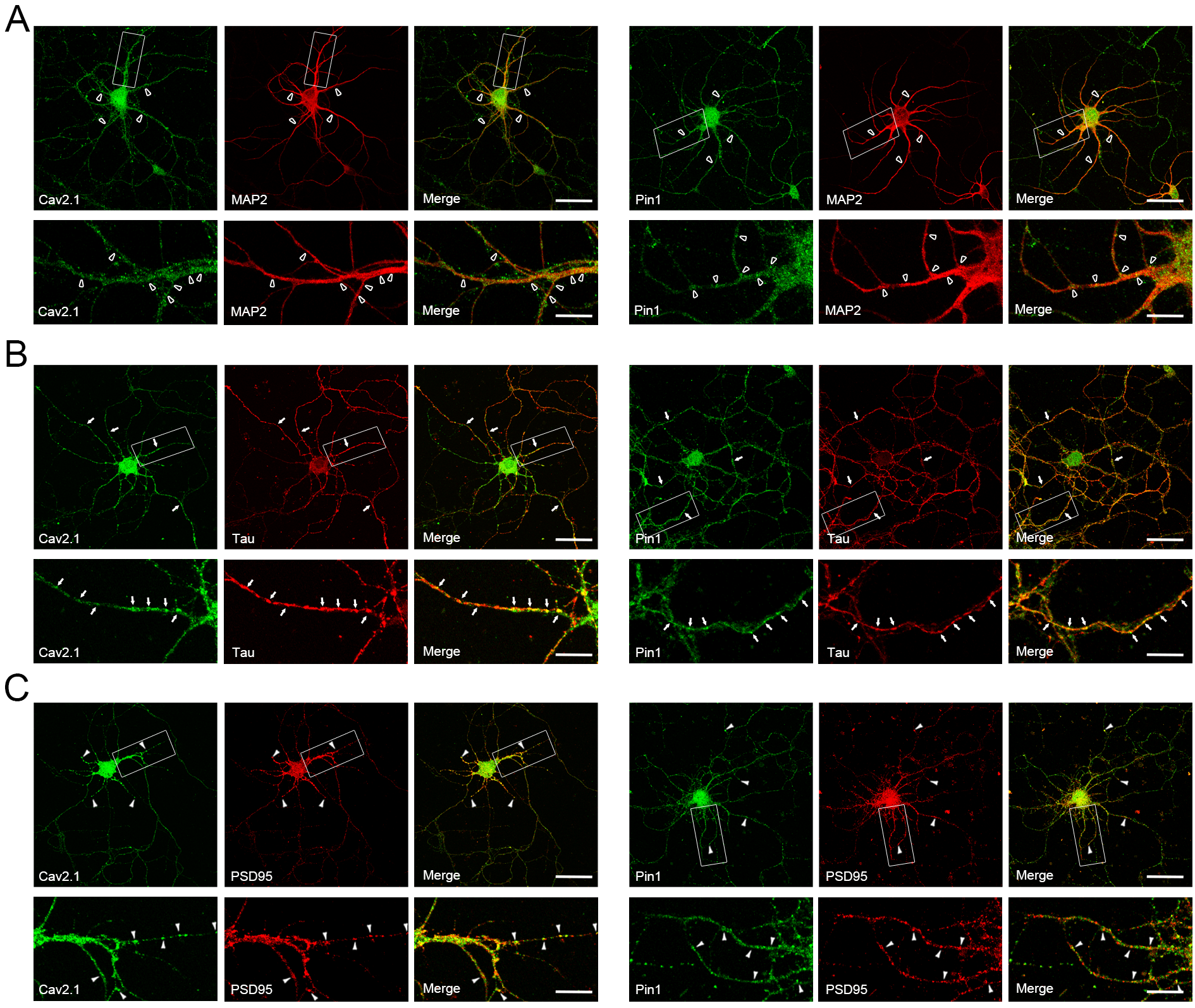

Supplement: Supplementary file 1 — Supplementary Material 1: Supplementary figure S1 Representative confocal images of endogenous CaV2.1 (left panels) and Pin1 (right panels) immunofluorescent signals in rat DIV10 cortical neurons. For each section, the boxed regions in the images shown in the upper rows are magnified for detailed inspection in the corresponding lower rows. A Localization of CaV2.1 or Pin1 (green) in MAP2-positive (red) dendrites and somas, as highlighted by open triangles and further demonstrated in the merge images. B Localization of CaV2.1 or Pin1 (green) in tau-positive (red) axons, as highlighted by arrows. C Colocalization of CaV2.1 or Pin1 (green) with PSD95-puncta (red) along neurites, as denoted by arrowheads and further highlighted by yellow puncta in the merge images. Scale bars, 50 μm (upper rows) and 12.5 μm (lower rows) [file 12964_2024_1960_MOESM1_ESM.tif]

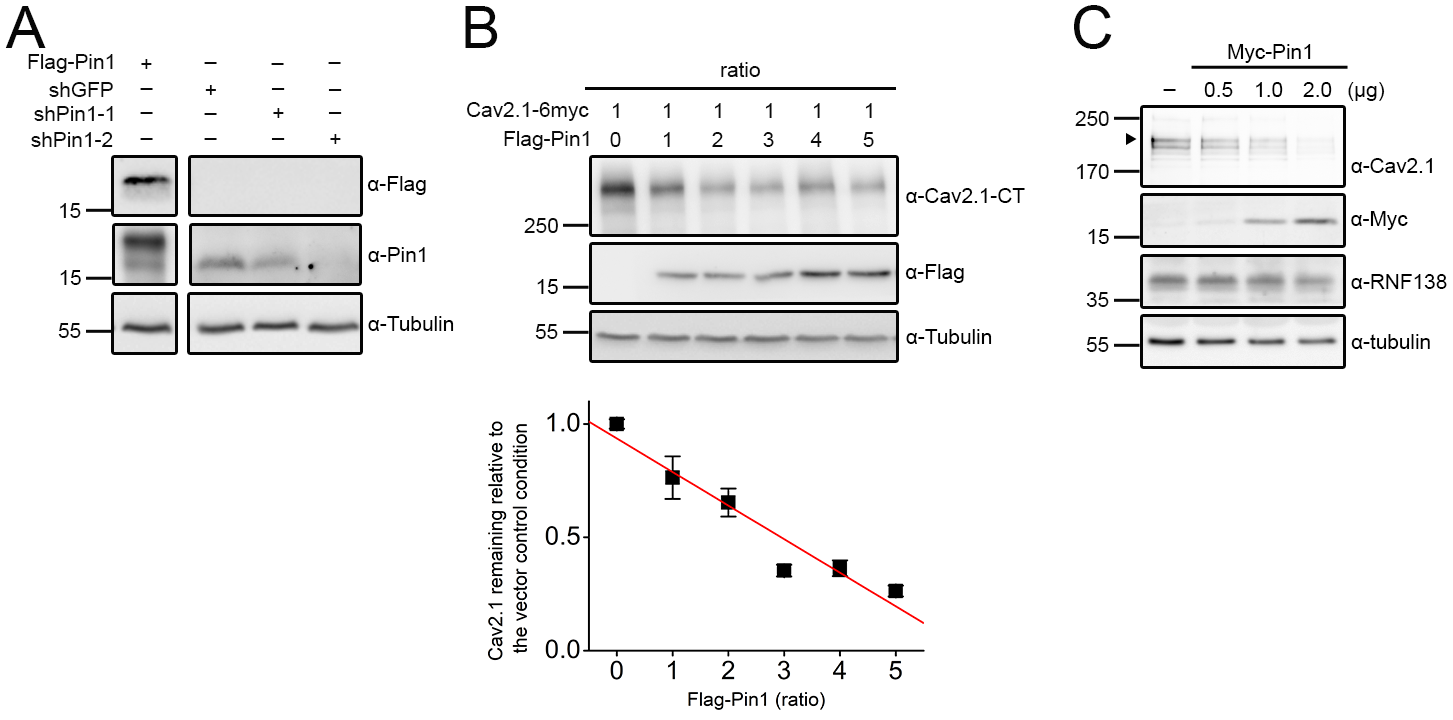

Supplement: Supplementary file 2 — Supplementary Material 2: Supplementary figure S2 Suppression of CaV2.1 protein level by Pin1 overexpression. A Representative immunoblots verifying the specificity of the anti-Pin1 antibody, as well as comparing the relative apparent molecular weights of Flag-tagged rat Pin1 (left panels) and endogenous human Pin1. HEK293T cells were subject to shGFP or shPin1 infection (right panels) to validate the protein band corresponding to endogenous Pin1. B (Top) Representative immunoblot comparing CaV2.1 protein levels in response to increasing coexpression ratios of Flag-Pin1 in HEK293T cells. The expression of tubulin is shown as the loading control. (Bottom) Quantification of relative CaV2.1 protein levels with respect to Pin1 co-transfection ratios. CaV2.1 signals were standardized as the ratio to the cognate tubulin signals, followed by normalization to the no Pin1 control, as well as single linear-regression analysis. Data were compiled from 3 independent experiments. C Representative immunoblot showing the effect of increasing the amount of Myc-Pin1 cDNA for transfection on endogenous CaV2.1 (arrowhead) protein levels in HT-22 mouse hippocampal cells. Also shown are endogenous RNF138 and tubulin expression [file 12964_2024_1960_MOESM2_ESM.tif]

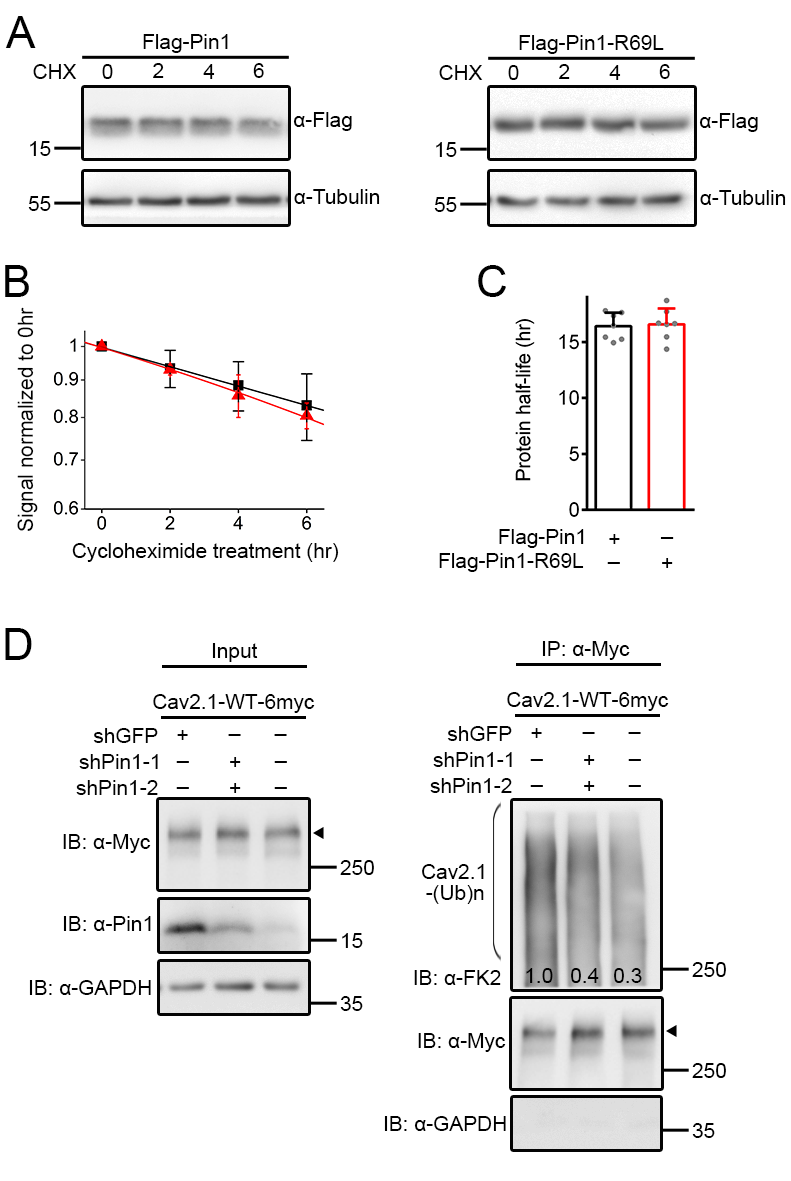

Supplement: Supplementary file 3 — Supplementary Material 3: Supplementary figure S3 Examination of Pin1 stability and the role of endogenous Pin1. A-C Protein turnover time course of Flag-tagged Pin1 and Pin1 R69L overexpressed in HEK293T cells. A Representative immunoblots. Transfected cells were subject cycloheximide (CHX) treatment for the indicated durations. B Quantification of Pin1 (black) and Pin1 R69L (red) protein turnover kinetics (n = 7). Protein density was normalized with respect to the corresponding value for no CHX treatment (0 h), followed by transformation into semilogarithmic plot and single linear-regression analyses. C Protein half-life values (hr): Pin1 (black), 16.40 ± 1.21; Pin1-R69L (red), 16.58 ± 1.41. D Representative immunoblots showing the effect of shRNA knockdown of endogenous Pin1 on human CaV2.1 polyubiquitination in HEK293T cells. shGFP was used as the control. Lysates from cells overexpressing CaV2.1-6myc were immunoprecipitated with α-Myc, followed by immunoblotting with the anti-ubiquitin antibody α-FK2. CaV2.1 polyubiquitination [CaV2.1-(Ub)n] by endogenous ubiquitin is visualized as high-molecular-weight protein smears. Normalized densitometric CaV2.1 ubiquitination intensity is labeled on the immunoblot. Corresponding expression level of CaV2.1, Pin1, and GAPDH in the lysates is shown in the Input lane [file 12964_2024_1960_MOESM3_ESM.tif]

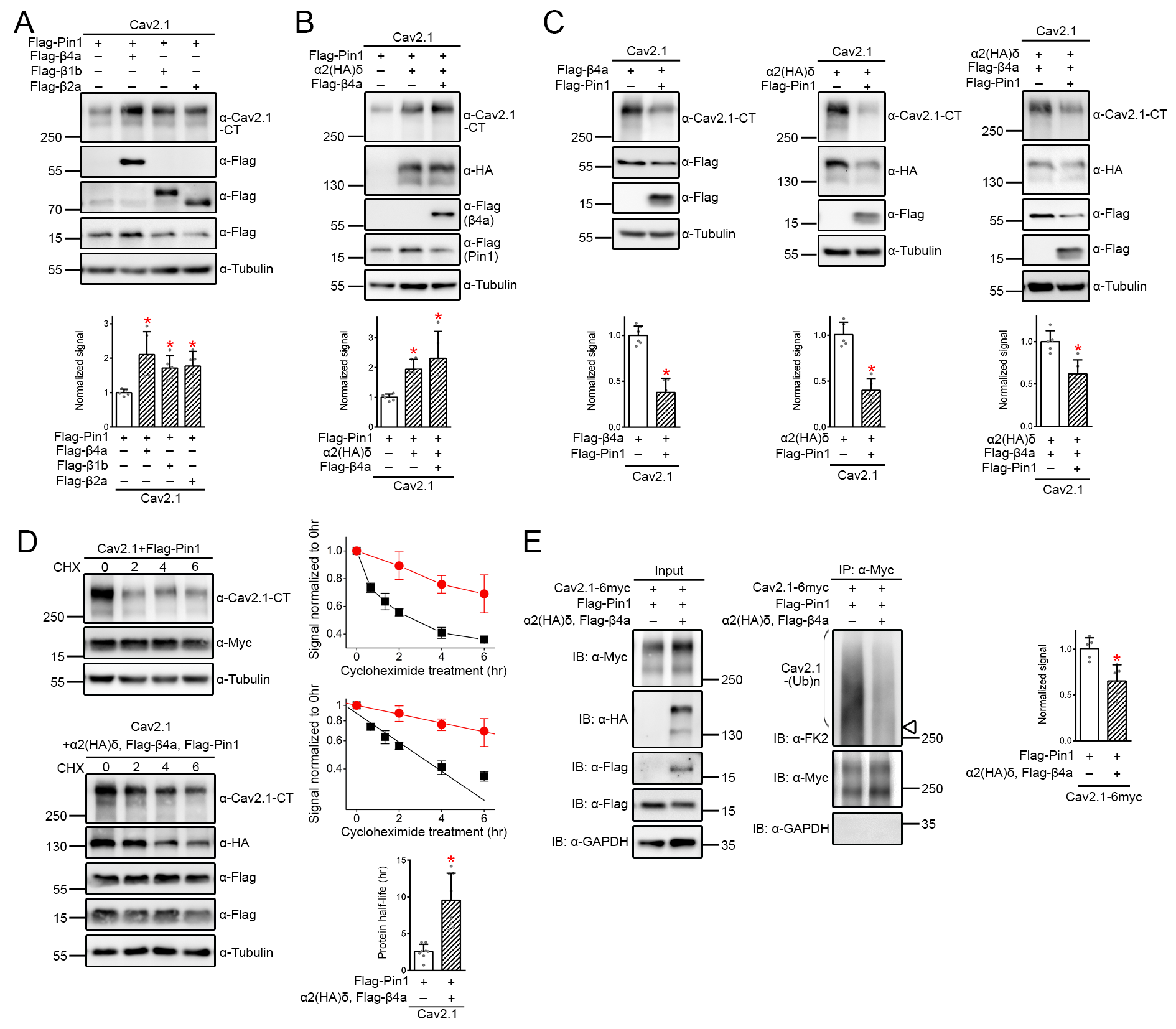

Supplement: Supplementary file 4 — Supplementary Material 4: Supplementary figure S4 Independent regulation of human CaV2.1 proteostasis by Pin1 and auxiliary subunits in HEK293T cells. A-B (Top Panels) Representative immunoblots showing the effect of the indicated auxiliary subunits on CaV2.1 regulation by Pin1. CaV2.1 was coexpressed with Pin1, α2δ, and β subunits in the molar ratios 1:3, 1:2, and 1:1, respectively. Coexpression with the Flag vector was used as the control. (Bottom Panels) Quantification of relative CaV2.1 protein level (n = 5–6). Data were normalized with respect to the corresponding Flag vector control: (A) Pin1 + vector, 1.00 ± 0.10; Pin1 + β4a, 2.10 ± 0.67; Pin1 + β1b, 1.71 ± 0.36; Pin1 + β2a, 1.77 ± 0.43. (B) Pin1 + vector, 1.01 ± 0.10; Pin1 + α2δ, 1.94 ± 0.33; Pin1 + α2δ-β4a, 2.31 ± 0.90. C (Top Panels) Representative immunoblots showing the effect of Pin1 on CaV2.1 regulation by the indicated auxiliary subunits. Coexpression with the Flag vector was used as the control. (Bottom Panels) Quantification of relative CaV2.1 protein level (n = 5–6). Data were normalized with respect to the corresponding Flag vector control: (left) β4a + vector, 1.00 ± 0.10; β4a + Pin1, 0.38 ± 0.15. (center) α2δ + vector, 1.01 ± 0.13; α2δ + Pin1, 0.40 ± 0.12. (right) α2δ-β4a + vector, 1.00 ± 0.13; α2δ-β4a + Pin1, 0.62 ± 0.16. D Representative immunoblots and quantification of the effect of α2δ-β4a subunits on Pin1 regulation of CaV2.1 protein stability. CaV2.1 protein half-life values (hr) (n = 8): Pin1 + vector (black), 2.57 ± 1.00; Pin1 + α2δ-β4a (red), 9.54 ± 3.68. E Representative immunoblots and quantification of the effect of α2δ-β4a subunits on Pin1 regulation of CaV2.1 polyubiquitination. Normalized CaV2.1 ubiquitination signal (n = 5): Pin1 + vector, 1.01 ± 0.12; Pin1 + α2δ-β4a, 0.65 ± 0.18. Asterisks denote significant difference from the cognate vector control (*, P < 0.05) [file 12964_2024_1960_MOESM4_ESM.tif]

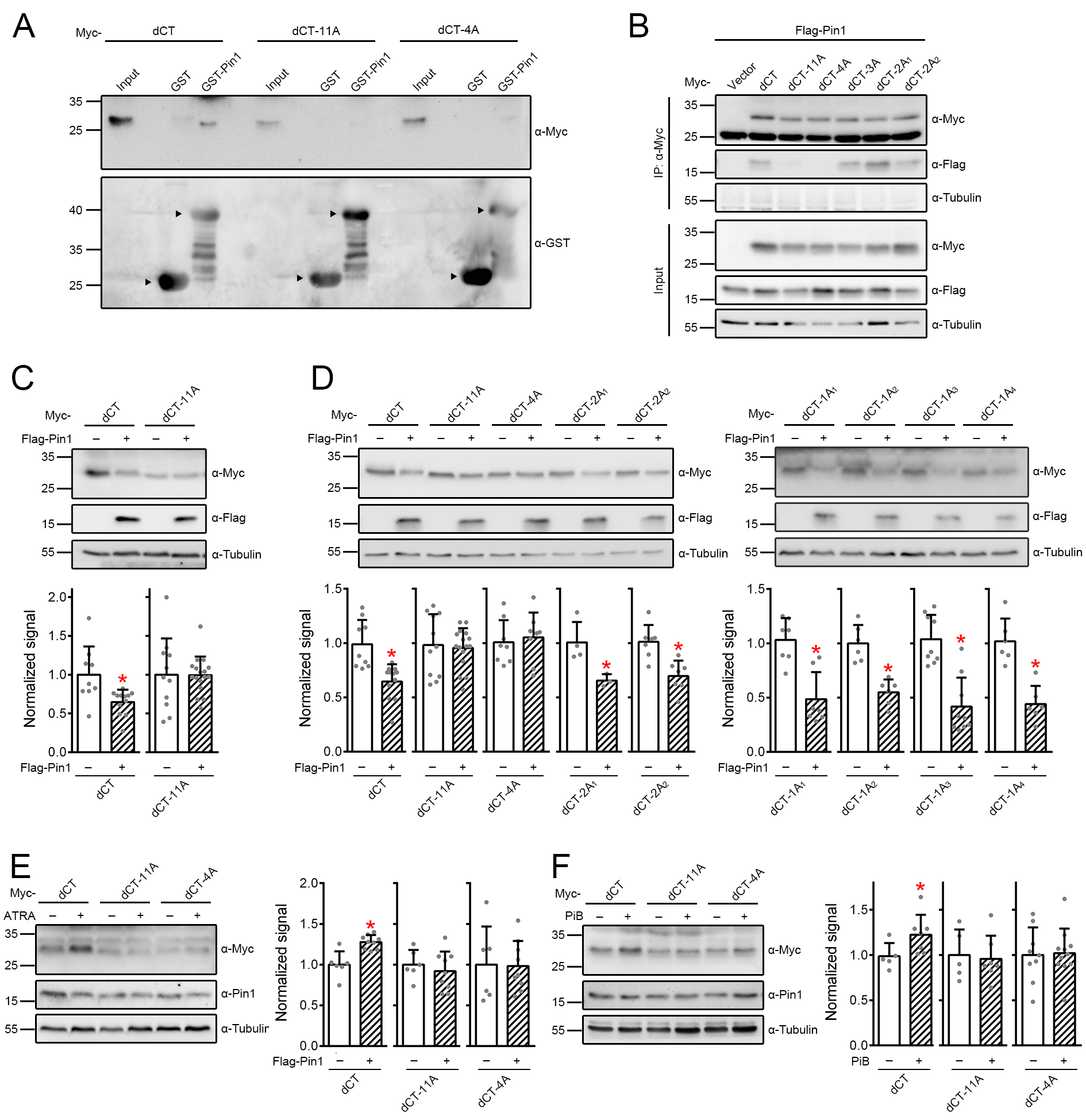

Supplement: Supplementary file 5 — Supplementary Material 5: Supplementary figure S5 Alanine mutation of potential Pin1-interacting serine/threonine residues in the distal carboxy-terminal (dCT) fragment of human CaV2.1 long-isoform. dCT: no alanine mutation was carried out in the dCT fragment. dCT-11 A: all 11 potential Pin1-interacting serine/threonine residues (see Fig. 5C) were mutated into alanine. dCT-4 A: Four potential Pin1-interacting serine/threonine residues (corresponding to S2254, S2274, T2284, and S2383 in Fig. 5C) were mutated into alanine. dCT-3/2/1A: three/two/one potential Pin1-interacting serine/threonine residues were mutated into alanine. A Representative immunoblots showing the lack of interaction between GST-Pin1 and Myc-tagged dCT fragments harboring the indicated alanine mutations. Lysates from HEK293T cells overexpressing various dCT constructs were subject to GST pull-down assay with GST or GST-Pin1, followed by immunoblotting with α-Myc and α-GST. Arrowheads denote the location of GST or GST-Pin1 fusion protein bands. B Representative immunoblots demonstrating deficient coimmunoprecipitation of Pin1 with dCT fragments harboring the indicated alanine mutations. Lysates from HEK293T cells coexpressing Flag-Pin1 with various Myc-dCT constructs were subject to immunoprecipitation with α-Myc, followed by immunoblotting with α-Myc and α-Flag. C-D Lack of effect of Pin1 on protein expression of dCT-4 A and dCT-11 A in HEK293T cells. (Top panels) Representative immunoblots depicting the effect of coexpression with Flag-Pin1. Coexpression with Flag vector (−) was used as the control. (Bottom panels) Quantification of relative dCT protein level (n = 3–17). Asterisks denote significant difference from the cognate vector control (*, P < 0.05). E-F Lack of effect of Pin1-suppressing ATRA (E) and PiB (F) on protein expression of dCT-4 A and dCT-11 A in HEK293T cells. (Left panels) Representative immunoblots displaying the effect of treatment with 50 µM ATRA (E) and 10 µM PiB (F). Treatm [file 12964_2024_1960_MOESM5_ESM.tif]

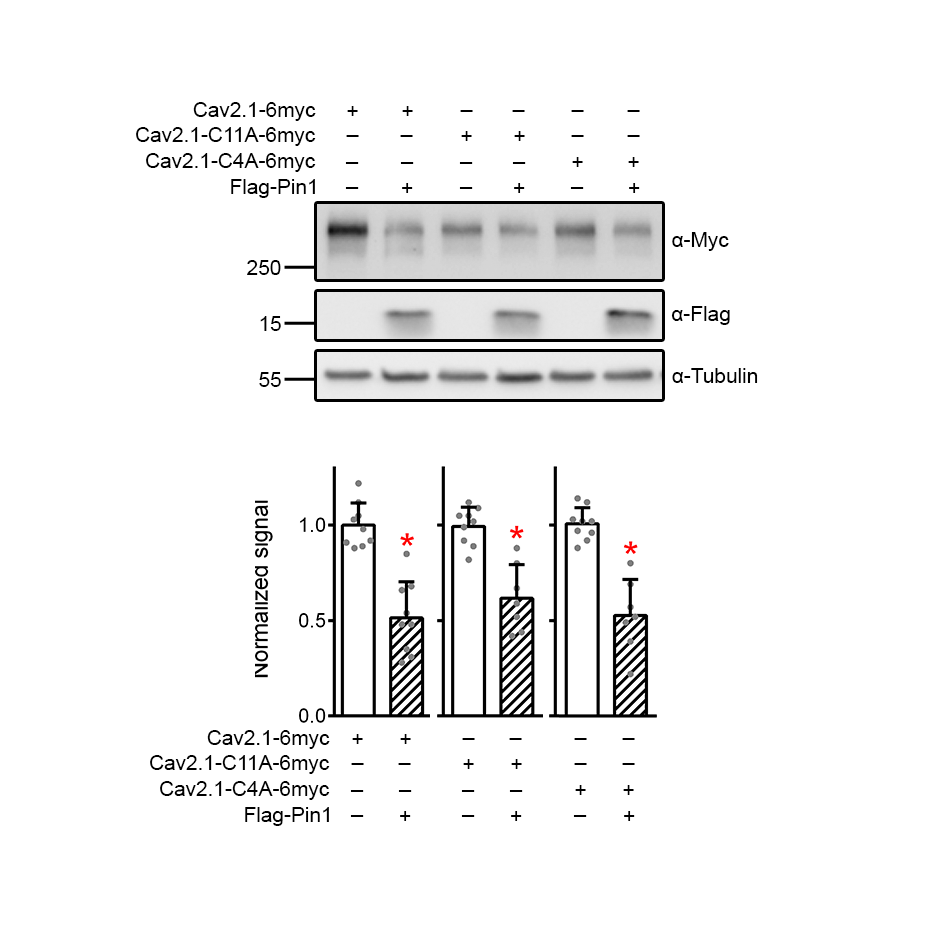

Supplement: Supplementary file 6 — Supplementary Material 6: Supplementary figure S6 Pin1 regulation of human CaV2.1 long-isoform harboring alanine substitution of either the 4 essential or all 11 distal carboxy-terminal Pin1-interacting serine/threonine residues (CaV2.1-C4A, CaV2.1-C11A). (Top panels) Representative immunoblots depicting the effect of coexpression with Flag-Pin1 on CaV2.1 in HEK293T cells. Coexpression with Flag vector (−) was used as the control. (Bottom panels) Quantification of relative protein level. Normalized CaV2.1 signal (n = 9): vector, 1.00 ± 0.12; Pin1, 0.51 ± 0.19. Normalized CaV2.1-C4A signal (n = 7–9): vector, 1.01 ± 0.09; Pin1, 0.53 ± 0.19. Normalized CaV2.1-C11A signal (n = 7–9): vector, 1.00 ± 0.10; Pin1, 0.62 ± 0.18. Asterisks denote significant difference from the cognate vector control (*, P < 0.05) [file 12964_2024_1960_MOESM6_ESM.tif]

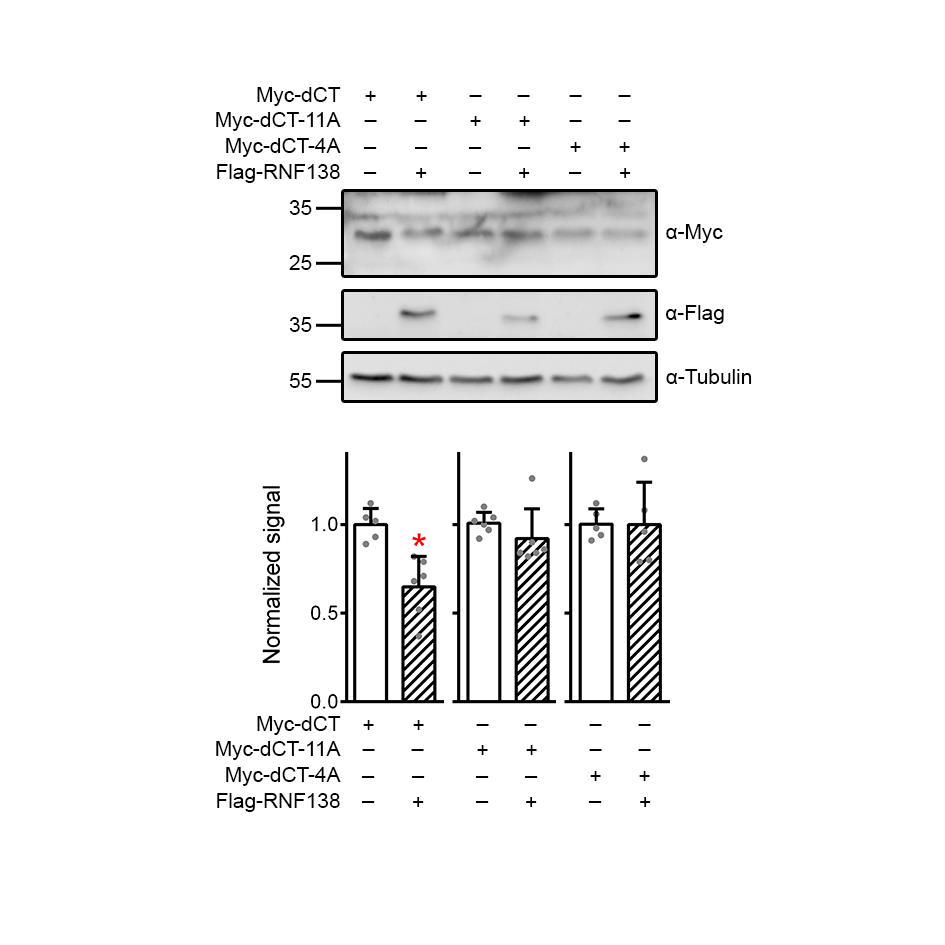

Supplement: Supplementary file 7 — Supplementary Material 7: Supplementary figure S7 Lack of effect of RNF138 on Pin1-insenstive human CaV2.1 dCT fragments harboring alanine substitution of either the 4 essential or all 11 Pin1-interacting serine/threonine residues (dCT-4 A, dCT-11 A). Representative immunoblots and quantification of the effect of RNF138 coexpression on the indicated dCT fragment constructs in HEK293T cells. Normalized dCT signal (n = 5–6): vector, 1.00 ± 0.09; RNF138, 0.65 ± 0.17. Normalized dCT-4 A signal (n = 5): vector, 1.00 ± 0.09; RNF138, 1.00 ± 0.24. Normalized dCT-11 A signal (n = 6): vector, 1.01 ± 0.06; RNF138, 0.92 ± 0.17. Asterisk denotes significant difference from the cognate vector control (*, P < 0.05) [file 12964_2024_1960_MOESM7_ESM.tif]

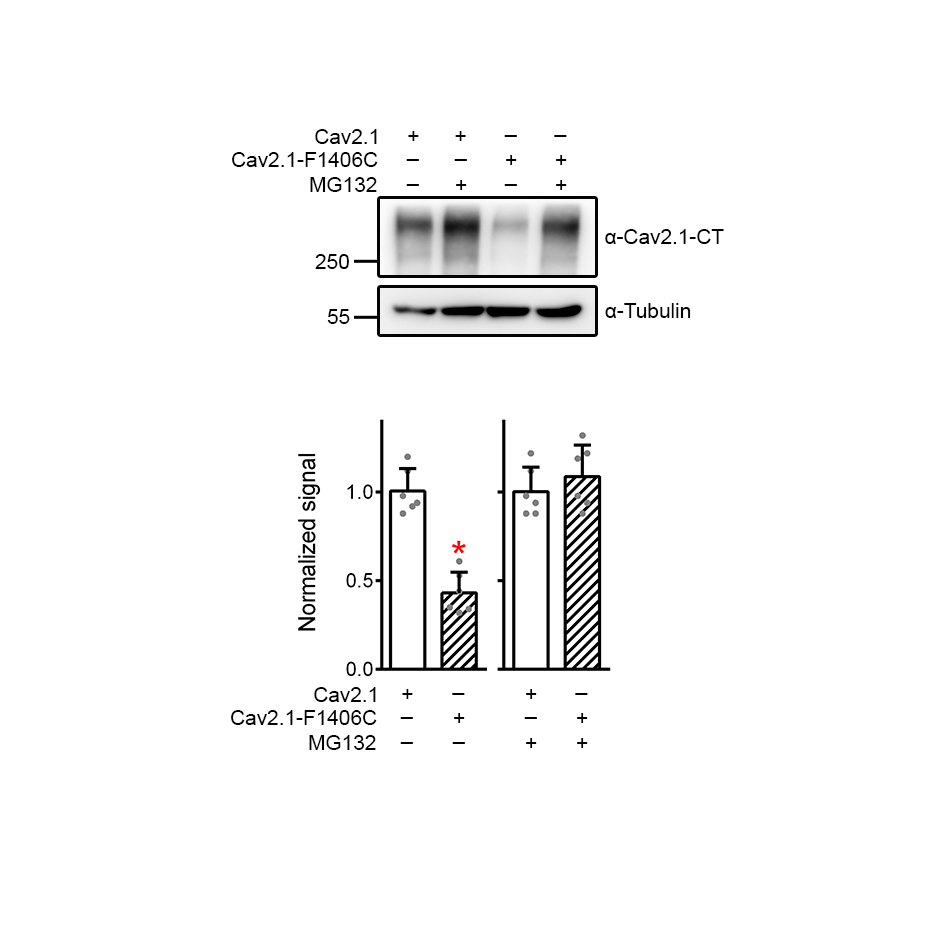

Supplement: Supplementary file 8 — Supplementary Material 8: Supplementary figure S8 Enhanced protreasomal degradation of CaV2.1-F1406C. Representative immunoblots and quantification of the effect of the proteasomal inhibitor MG132 on relative protein expression of CaV2.1 and CaV2.1-F1406C. Lysates from transfected HEK293T cells were subject to treatment with DMSO or 10 µM MG132 for 24 h. Normalized protein level in response to DMSO (n = 6): CaV2.1, 1.01 ± 0.13; CaV2.1-F1406C, 0.43 ± 0.12. Normalized protein level in response to MG132 (n = 6): CaV2.1, 1.01 ± 0.14; CaV2.1-F1406C, 1.09 ± 0.18. Asterisk denotes significant difference from CaV2.1 (*, P < 0.05) [file 12964_2024_1960_MOESM8_ESM.tif]

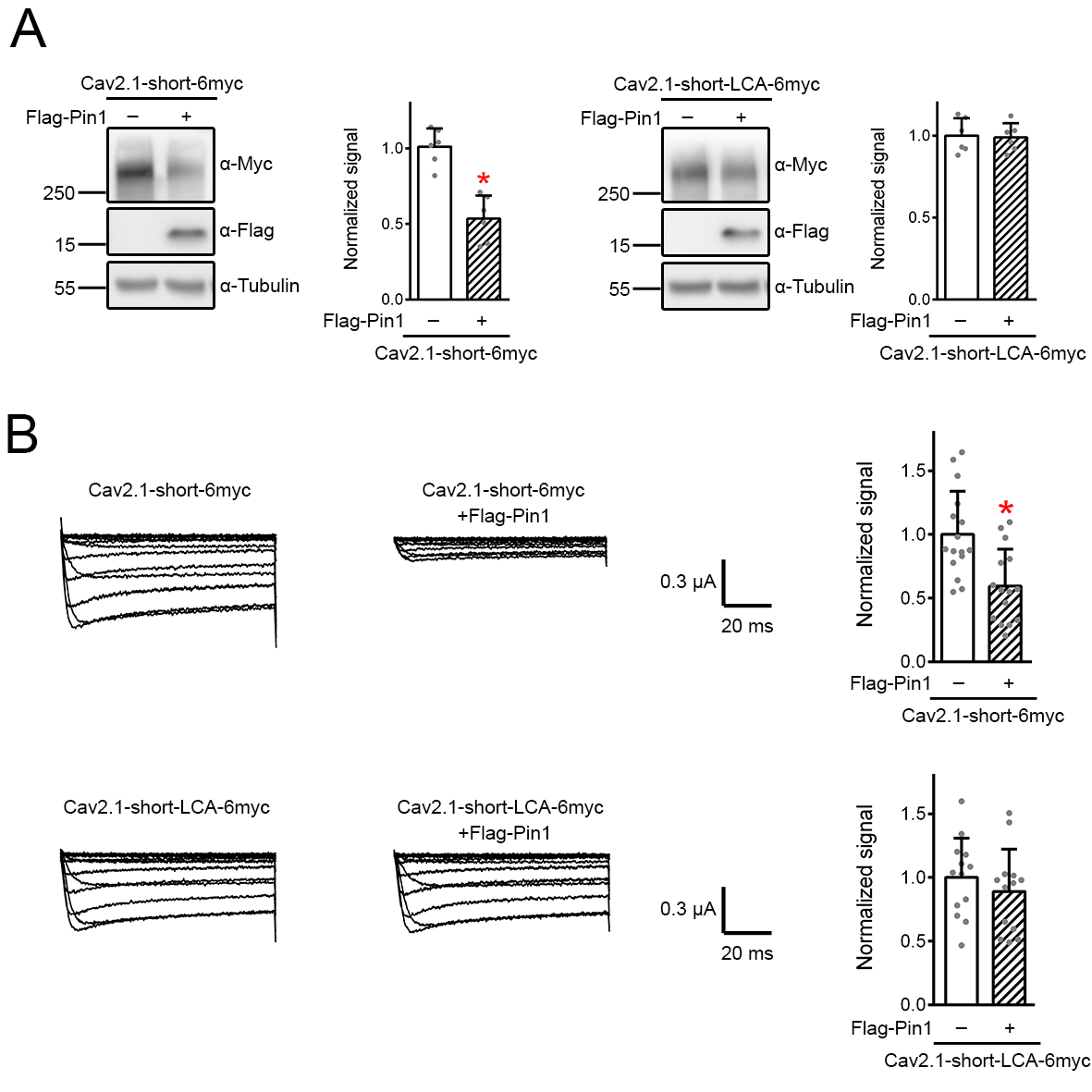

Supplement: Supplementary file 9 — Supplementary Material 9: Supplementary figure S9 Lack of effect of Pin1 on the human CaV2.1 short-isoform construct harboring alanine substitution of all the potential Pin1-interacting serine/threonine residues in the II-III loop and the distal carboxy-terminal region (CaV2.1-short-LCA). A Representative immunoblots and quantification of the effect of Pin1 on protein level of CaV2.1-short and CaV2.1-short-LCA in HEK293T cells. Data were normalized with respect to the corresponding vector control. Normalized CaV2.1-short signal (n = 6): vector, 1.01 ± 0.12; Pin1, 0.54 ± 0.15. Normalized CaV2.1-short-LCA signal (n = 6): vector, 1.00 ± 0.11; Pin1, 0.99 ± 0.09. Asterisk denotes significant difference from the cognate vector control (*, P < 0.05). B Representative Ba2+ current traces and quantification of the effect of Pin1 on functional expression of CaV2.1-short and CaV2.1-short-LCA in Xenopus oocytes. Data were normalized with respect to the corresponding water coinjection control. Normalized CaV2.1-short current amplitude at + 20 mV (n = 15–16): control, 1.00 ± 0.34; Pin1, 0.59 ± 0.29. Normalized CaV2.1-short-LCA current amplitude at + 20 mV (n = 13): control, 1.00 ± 0.31; Pin1, 0.89 ± 0.33. Asterisk denotes significant difference from the cognate control (*, P < 0.05) [file 12964_2024_1960_MOESM9_ESM.tif]
